# A reference genome for cultured Drosophila ovarian somatic cells enables studies of transposon and piRNA biology

**DOI:** 10.1186/s13059-026-04113-y

**Published:** 2026-05-22

**Authors:** Dominik Handler, Julius Brennecke

**Affiliations:** Institute of Molecular Biotechnology of the Austrian Academy of Sciences (IMBA), Vienna BioCenter (VBC), Vienna, 1030 Austria

## Abstract

**Background:**

Accurate reconstruction of repetitive genomic regions is essential to understand small RNA–mediated transposon silencing. In Drosophila ovarian somatic cells (OSCs), a primary model for nuclear PIWI–piRNA biology, reliance on the D. melanogaster reference (dm6) obscures cell-line–specific transposon insertions and the architecture of piRNA source loci.

**Results:**

We generated a highly contiguous de novo assembly of the OSC genome that reveals a transposon landscape diverging substantially from dm6. The assembly fully resolves the ∼700 kb flamenco locus, the principal piRNA cluster in OSCs, and yields a genome-wide map of transposon insertions. By integrating this assembly with functional genomics datasets, we define OSC piRNA source loci, show that cluster sequence composition determines transposon piRNA output, and demonstrate the widespread effects of Piwi on heterochromatin formation.

**Conclusions:**

The OSC assembly establishes a precise genomic framework for interpreting piRNA and chromatin data in this model system. To enable community use of this resource for studies of transposon control and piRNA biology, we provide an integrated genome browser and data-sharing platform.

**Supplementary Information:**

The online version contains supplementary material available at 10.1186/s13059-026-04113-y.

## Background

Transposable elements are mobile genetic elements that occupy substantial portions of eukaryotic genomes and threaten genome stability [1–3]. Across plants, fungi, and animals, transposon activity is restrained by small RNA-based silencing systems that utilize Argonaute proteins to repress transposon expression at both transcriptional or post-transcriptional levels [4–6]. The specificity of these pathways is determined by Argonaute-bound small RNAs, which are predominantly derived from transposon-rich genomic regions.

Because most small RNAs involved in genome defense originate from repetitive loci, they map to multiple genomic locations. This inherent ambiguity complicates the reconstruction of their genomic origins and obscures their functional relationships with active transposons. Custom genome assemblies tailored to specific experimental systems mitigate these challenges by capturing the precise transposon landscape, which often differs considerably from standard reference genomes. By resolving unique insertion sites and the complex architecture of small RNA source loci, these assemblies provide the necessary framework to study small RNA biogenesis and targeting with high resolution.

In animals, genome defense is primarily mediated by the PIWI-interacting RNA (piRNA) pathway, a conserved mechanism operating from sponges to mammals. This pathway functions predominantly in the gonads to silence transposons within both germ cells and the surrounding somatic tissues [7–9]. The fruit fly, *Drosophila melanogaster*, serves as a leading model for piRNA research, supported by a sophisticated genetic toolkit and long history of transposon discovery [10, 11]. A key experimental system is the ovarian somatic cell (OSC) line, one of the few animal cell lines that maintains a functional, transposon-repressing piRNA pathway [12, 13].

The OSC piRNA pathway mirrors that of the ovarian follicle cells and relies on a single nuclear PIWI-clade Argonaute, Piwi [13–16]. Unlike germ cells, OSCs lack the cytoplasmic PIWI proteins Aubergine and Ago3 and do not utilize the ping-pong piRNA amplification cycle [17, 18]. Instead, single-stranded piRNA precursors enriched in transposon antisense sequences are processed on the mitochondrial surface into phased 23–32 nt piRNAs and loaded into Piwi [7–9]. Upon nuclear import [19], the Piwi-piRNA complex recognizes nascent transposon transcripts via sequence complementarity. This engagement triggers the assembly of the Piwi* complex—including Asterix/Gtsf1 and Maelstrom—which recruits heterochromatin factors to enforce transcriptional silencing [20–30].

The streamlined nature of the OSC system, featuring a single Argonaute and a linear piRNA biogenesis pathway, makes it an ideal model for dissecting phased piRNA biogenesis and piRNA-guided heterochromatin formation. However, despite its widespread use, the genomic architecture of the OSC line remains poorly defined. To date, studies have relied on the *Drosophila melanogaster* reference genome (dm6) [17, 31, 32]. While dm6 resolves significant portions of repeat-rich heterochromatin, it represents a fly strain that differs markedly from the laboratory strains used to establish the OSC line [33]. Furthermore, cell line immortalization often involves extensive genomic rearrangements and bursts of transposon activity, creating novel insertions absent from the reference. Indeed, short-read DNA sequencing has indicated that the OSC transposon landscape diverges significantly from dm6 [24, 33]. These discrepancies hinder accurate piRNA mapping, cluster annotation, and the interpretation of chromatin profiles, ultimately limiting the precision of OSC-based research.

Here, we present a high-quality de novo assembly of the OSC genome with scaffold continuity comparable to the *Drosophila melanogaster* reference. Our assembly includes all major piRNA source loci, including a complete reconstruction of the rapidly evolving *flamenco* cluster. In addition, it provides a comprehensive, genome-wide map of transposon insertions and reports on structural variants and single nucleotide polymorphisms, facilitating the design of effective siRNAs and guide RNAs. Together, these features establish a precise genomic framework for interpreting piRNA and chromatin datasets at nucleotide resolution. To ensure broad accessibility, we provide the assembly in standard formats and as an interactive UCSC Genome Browser hub for seamless integration with user-generated data.

## Results & discussion

### A high-quality de novo assembly of the OSC genome

The *Drosophila* ovarian somatic cell (OSC) line was established in 2009 as a stable derivative of the mixed fGS/OSS culture, which originated from *bam* mutant ovaries [12, 13, 33]. We obtained the OSC line from the Siomi laboratory in 2010, established a master cell bank, and utilized an early passage to isolate high-molecular-weight genomic DNA. Oxford Nanopore Technologies (ONT) long reads (~100x coverage) and Illumina short reads (~50x coverage) provided the depth necessary for a highly contiguous genome assembly.

**Fig. 1:**
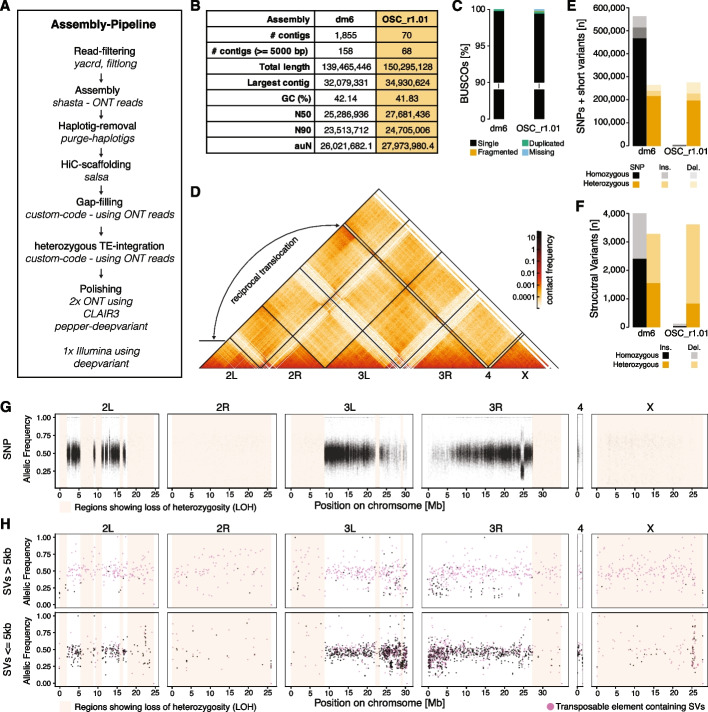
A highly contiguous OSC genome assembly. **A** Assembly workflow integrating Oxford Nanopore (ONT) long reads, Illumina short reads, and Hi-C data. **B** Assembly statistics comparing the dm6 reference genome and the OSC_r1.01 genome. **C** BUSCO completeness comparison between dm6 and OSC_r1.01. **D** Hi-C contact frequency map of the OSC_r1.01 assembly revealing a heterozygous translocation of distal 2L to 3R. **E** Homozygous and heterozygous SNPs and small variants identified by DeepVariant from Illumina reads obtained from OSC genomic DNA aligned to the dm6 or the OSC_r1.01 assemblies. **F** Homozygous and heterozygous structural variants (SVs) detected by Sniffles from ONT reads obtained from OSC genomic DNA aligned to the dm6 or the OSC_r1.01 assemblies. **G** Genome-wide distribution of SNP allele frequencies (based on Illumina reads) across chromosomes in the OSC_r1.01 assembly. **H** Genome-wide distribution of short (<5 kb, top) and long (=>5 kb, bottom) SV frequencies (based on ONT reads) across chromosomes in the OSC_r1.01 assembly. Shaded areas in G, and H, indicate regions showing loss of heterozygosity

To assemble the OSC genome, we developed a multi-step pipeline optimized for the recovery of transposable element sequences (Fig. 1A). The integration of Hi-C chromosome conformation capture data further refined scaffold continuity and chromosome-level organization. A significant challenge was the non-isogenic nature of the OSC genome, particularly regarding heterozygous transposon insertions and structural variants. Rather than generating separate haplotype genomes, we generated a consensus genome where transposon insertions and structural variants were retained by default, and heterozygous SNPs were randomly selected but fully documented (Fig. 1A).

Because heterozygosity in cell culture can reflect either true diploid allelic variation or sub-population mosaicism, we analyzed DNA-seq data from a recently derived clonal OSC line, established from a single cell [34], to determine if heterozygous features were fixed. This analysis revealed that the vast majority of transposon insertions classified as heterozygous in the assembly are present in the clonal line, confirming they are stable, fixed features of the OSC genome (Additional file 1: Fig. S1A, B).

The final assembly (OSC_r1.01) spans ~150 Mb and surpasses the current *Drosophila melanogaster* reference genome (dm6) across most continuity metrics (Fig. 1B, Additional file 1: Fig. S1C). Even when scaffolds are broken into contigs, continuity remains higher than for dm6 (Additional file 1: Fig. S1D). Benchmarking Universal Single-Copy Orthologs (BUSCO) analysis confirmed that the OSC genome is also highly complete, comparable in quality to the reference genome (Fig. 1C). While highly contiguous, the assembly is not yet telomere-to-telomere; as with other *Drosophila* assemblies, extremely repetitive regions—including telomeric transposon arrays, the histone gene cluster, rDNA repeats, and centromeres—remain only partially resolved.

To assess structural integrity, we mapped Hi-C reads obtained from OSCs onto the assembled genome (Fig. 1D). This analysis confirmed the overall assembly quality while revealing a complex, heterozygous reciprocal translocation between the distal ~10 Mb of chromosome 2L and the terminus of chromosome 3R (Fig. 1D, Additional file 1: Fig. S1E). Long Nanopore reads supported this rearrangement on one homolog and identified accompanying small segmental duplications at both breakpoints, resulting in two distinct 2L–3R junctions (Additional file 1: Fig. S1F, G). The breakpoints disrupt one copy each of *Oatp30B* (2L) and *heph* (3R) on the translocated homolog.

At the nucleotide level, we benchmarked the assembly by calling single nucleotide polymorphisms (SNPs), short variants smaller than 50 bp, and structural variants above 50 bp (Fig. 1E, F). Re-mapping OSC-derived Illumina and Nanopore reads to our assembly yielded fewer than 6,000 homozygous aberrations. In contrast, mapping the same reads to dm6 revealed over 500,000 homozygous differences, highlighting considerable differences between the two genomes. On average, the OSC genome differs from dm6 by ~ 6 SNPs per kb, which has important implications for experimental design. For example, the gene coding for the piRNA biogenesis factor SoYb harbors 85 homozygous nucleotide differences across its 4,431 bp coding sequence, highlighting the necessity of a cell-line-specific reference for designing effective siRNAs or CRISPR guide RNAs.

We next examined allelic variation to assess the heterozygosity of the OSC line. Consistent with its non-isogenic origin, we detected >250,000 heterozygous SNPs (Fig. 1E). However, these variants were unevenly distributed: SNP density plots along the chromosome arms revealed extensive ‘SNP deserts’, where density dropped from ~6 per kb to <0.1 (Fig. 1G; Additional file 1: Table S1). These regions correspond precisely to previously described loss-of-heterozygosity (LOH) domains, thought to have arisen from segmental deletions or copy-neutral mitotic recombination events during cell line immortalization [33]. Mapping of short and long structural variants showed that while short variants were also largely absent from LOH regions, long variants were still present (Fig. 1H). Consistent with previous reports [33], many long variants correspond to transposable element insertions, suggesting that new transposition events occurred after the LOH-generating episodes that shaped the immortalized OSC genome.

In summary, OSC_r1.01 is a high-quality, contiguous, and complete genomic resource. By providing detailed insights into the stable structural and allelic variation of this line, it establishes a robust foundation for high-resolution studies of transposon biology and piRNA-mediated genome defense.

### Annotation and genome browser integration of the OSC genome

**Fig. 2:**
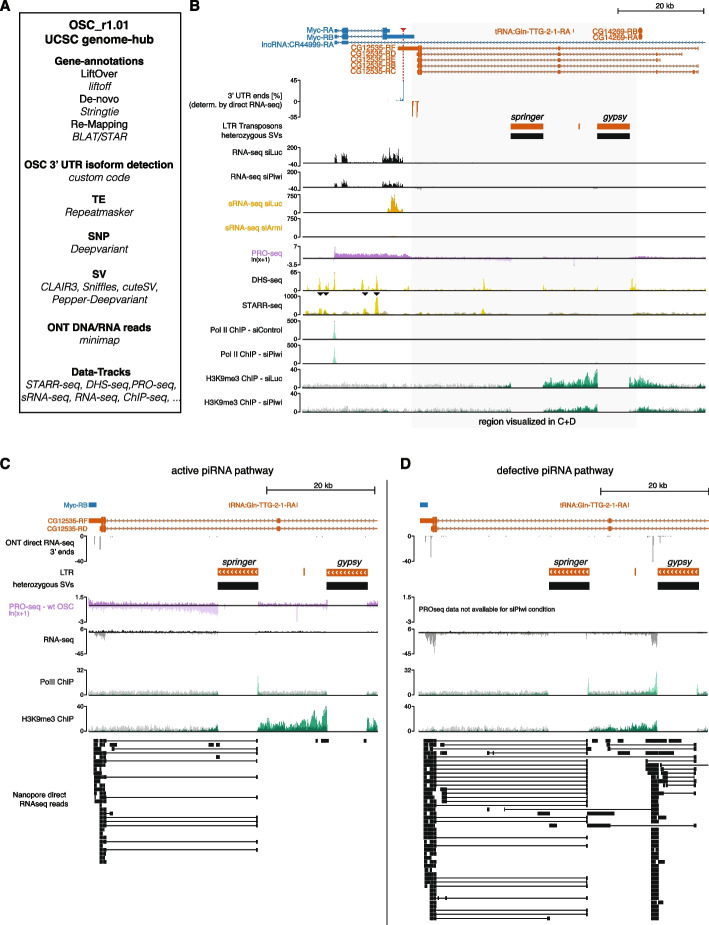
An OSC genome browser resource with functional gene expression datasets. **A** Overview of datasets and genome annotations included in the UCSC OSC genome browser with tools used for track generation or data type indicated. **B** Genome browser view of the *Myc* and *CG12535* loci. The extent of a novel 3′ UTR isoform of the *Myc* transcript identified by Nanopore direct RNA-seq 3′ end quantification is marked by a red arow. Individual detected genic 3′ ends are shown as a percentage of all detected 3′ ends for each gene. (genome-uniquely mapping STARR-seq, DHS-seq, ChIP-seq, PRO-seq and RNA-seq profiles are shown as coverage per million reads, small RNA coverage was normalized to 1 Mio miRNA reads, data is displayed as the average of 3 replicates; negative read-counts indicate coverage on the antisense strand; PRO-seq signal is displayed as ln(x + 1)-transformed coverage; ChIP-seq signal is shown as an overlay of ChIP (green) and ChIP input (grey); STARR-seq and DHS-seq are shown as an overlay of experiment (yellow) and input (grey); STARR-seq peaks in the *Myc* locus are indicated with black arrows). **C**, **D** Genome browser views of the *CG12535* locus containing heterozygous *springer* and *gypsy* LTR retrotransposon insertions. Shown in addition to (**B**) are ONT direct RNA-seq reads. **C** Control cells with an active piRNA pathway. **D** Piwi-depleted cells with a defective piRNA pathway (representative subset of ONT direct RNA-seq reads shown)

To ensure broad usability, we comprehensively annotated both genes and repeats in the OSC genome (Fig. 2A). Gene models from FlyBase [35] were aligned to the assembly, while transposable elements were annotated using RepeatMasker with a library of *Drosophila melanogaster* transposon consensus sequences [36]. Together, these annotations provide a detailed map of the gene and transposon landscape in OSCs.

To enrich these annotations, we integrated a diverse collection of published and newly generated sequencing datasets (Additional Table 2). This panel includes transcriptome data (poly(A)-selected and rRNA-depleted RNA-seq, small RNA-seq, direct long read RNA-seq), transcriptional dynamics (precision run-on (PRO)-seq, Pol II chromatin-immunoprecipitation (ChIP)-seq), chromatin state (ChIP-seq for H3K9me3, DNase-hypersensitivity (DHS)-seq for chromatin accessibility), and enhancer activity (self-transcribing active regulatory region (STARR)-seq for developmental genes; [37]). To maximize accessibility, all annotations and functional datasets were organized into a dedicated UCSC genome browser hub (Additional file 1: Fig. S2A) [38]. This interactive platform enables users to explore the OSC genome at nucleotide resolution, compare functional data sets with FlyBase annotations, and integrate their own data.

To illustrate the utility of the OSC genome browser, we examined a 90-kb region on the distal X chromosome encompassing the *Myc* and *CG12535* loci (Fig. 2B). For *Myc*, PRO-seq and Pol II ChIP-seq revealed robust transcription from a single promoter, likely regulated by four distinct enhancers identified by STARR-seq. Direct RNA-seq showed that *Myc* produces a single transcript isoform in OSCs with a 3′ UTR that is distinct from FlyBase annotations (Additional file 1: Fig. S2B). This transcript is a major source of piRNAs that map almost exclusively to the 3′ UTR (Fig. 2B).

By contrast, *CG12535* exhibited no detectable promoter activity, yet RNA-seq revealed expression of its terminal two exons (Fig. 2B). Closer inspection identified a heterozygous insertion of a *springer* LTR retrotransposon within the second intron as the source of this expression. The *springer* element is oriented in the same direction as the host gene and is not targeted by antisense piRNAs in OSCs. Genome-uniquely mapping Pol II ChIP-seq and PRO-seq reads confirmed that the insertion is transcriptionally active (Fig. 2C). Moreover, direct RNA-seq demonstrated that transcription initiates at the first *springer* LTR and, through use of the *envelope* splice site donor [39], splices into the last two *CG12535* exons to produce transposon-mRNA chimeric transcripts (Fig. 2C; similar chimeric transcripts have also been described in [40]).

Notably, the *CG12535* locus also harbors a heterozygous insertion of the *envelope*-encoding *gypsy* retrovirus. In control cells, this element is embedded within H3K9me2/3-marked heterochromatin and remains transcriptionally silent (Fig. 2C), consistent with abundant *gypsy* antisense piRNAs in OSCs. Loss of Piwi-dependent heterochromatin derepressed the *gypsy* insertion (Pol II ChIP-seq) and indirectly also increased transcription from the neighboring *springer* element, resulting in elevated levels of *springer*-*CG12535* chimeric transcripts (RNA-seq, direct RNA-seq) (Fig. 2D).

Together, the OSC genome assembly and its integrated functional datasets establish a powerful framework to interrogate the interplay between transposons, gene regulation, and piRNA-mediated silencing, providing both a reference resource and a platform for new discoveries.

### The OSC transposon landscape and its regulation by the piRNA pathway

**Fig. 3:**
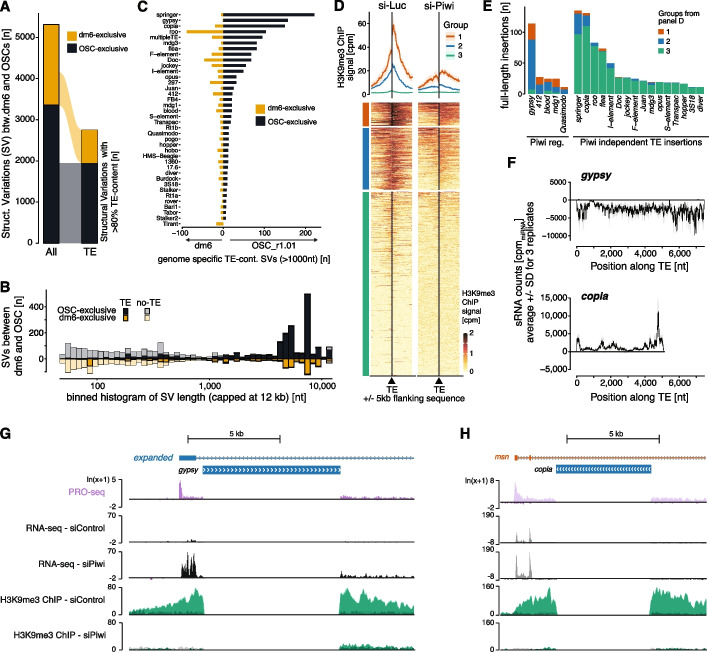
The OSC transposon landscape and its regulation by the piRNA pathway. **A** Quantification of structural variants (SVs) between OSC_r1.01 and dm6. Data are presented for all SVs (left) and for SVs comprising >80% transposable element content (right). Color grouping indicates the genome containing the additional sequence content. **B** Length distribution of the SVs determined in (**A**). SVs containing <80% transposable element content are shaded. **C** Structural variants (SVs) containing >80% transposon content, shown per element (minimum 10 variants; ‘multiple TE’ indicates SVs containing different transposon annotations). For each transposon, dm6-specific insertions are displayed to the left and OSC-specific insertions to the right. **D** Heatmaps of H3K9me3 ChIP-seq signal ±5 kb around full-length (>90% of consensus length) OSC transposon insertions in control (left) and *piwi*-depleted (right) conditions. Insertions were clustered into three groups by k-means, and the average ChIP-seq signal for each group is shown as a meta-profile above the heatmaps. **E** Classification of individual insertions from indicated transposons to the three groups defined in (**D**). Transposons were classified into *Piwi*-regulated or *Piwi*-independent categories based on an RNA-seq log₂ fold-change cutoff of >2.5. **F** Histogram plots showing piRNA coverage along the length of the *gypsy* and *copia* transposons. The x-axis indicates the position on the element, the y-axis shows average piRNA counts from three replicates, normalized to 1 million miRNAs (plus indicates sense piRNAs, minus indicates antisense piRNAs; shaded areas represent the standard deviation across replicates). **G, H** Genome browser views of the *expanded* (**F**) and *msn* (**H**) loci (ChIP-seq, PRO-seq and RNA-seq signals are shown as coverage per million reads; PRO-seq signal is displayed as ln(x + 1)-transformed coverage; negative read-counts indicate genomic minus strand reads; ChIP-seq is shown as an overlay of ChIP signal (green) and ChIP input (grey))

Through their inherent mobility, transposable elements generate significant structural variation. A whole-genome comparison between the OSC_r1.01 assembly and the dm6 reference revealed more than 5,000 structural variants larger than 50 bp (Fig. 3A). While most variants represent small insertions or deletions, a prominent class spanning 5–10 kb consists almost entirely of transposon insertions (Fig. 3B).

Most transposon-related structural variants in OSC_r1.01 are absent from the dm6 reference (Fig. 3C, Additional file 1: Fig. S3A). While these will include pre-existing germline variants from the *bam*-mutant founder strain, several lines of evidence suggest that a significant fraction represents novel mobilizations occurring during or after OSC immortalization [33]. Specifically, we identified numerous heterozygous transposon insertions within the large loss-of-heterozygosity (LOH) blocks in the OSC genome (see Fig. 1F). Because these segments were homogenized during cell line establishment, these heterozygous insertions must represent post-LOH transposition events. A few families of transposons disproportionately contributed to these insertions, including *springer*, *copia*, *gypsy*, and *flea* (Fig. 3C; Additional file 1: Fig. S3B). In contrast, families such as *roo* show numerous differences relative to dm6 but are virtually absent from the pool of heterozygous insertions in LOH regions, suggesting their distribution primarily reflects the founder strain's landscape. Furthermore, *Tirant*, an endogenous retrovirus that invaded natural *D. melanogaster* populations around the 1950 s [41], is abundant in dm6 (>17 full-length insertions) but entirely absent in OSC_r1.01, indicating the *bam*-mutant founder strain was *Tirant*-naïve (Fig. 3C).

The OSC line expresses a functional nuclear Piwi/piRNA pathway, where Piwi orchestrates the formation of H3K9me2/3-marked heterochromatin by binding piRNA-complementary nascent transposon transcripts [24]. With an accurate transposon insertion map, we assessed the heterochromatin status flanking all euchromatic transposon insertions using H3K9me3 ChIP-seq data. Hierarchical clustering grouped insertions into three categories: those associated with strong Piwi-dependent heterochromatin (group 1), those with weaker but still Piwi-dependent domains (group 2), and those with little or no heterochromatin (group 3) (Fig. 3D). This epigenetic landscape is remarkably stable; comparison of our 2023 data [34] with H3K9me3 ChIP-seq data generated in 2012 by us [24] or in 2016 by the Saito laboratory [42] revealed nearly identical profiles across all three groups (Additional file 1: Fig. S3C). Analysis of our clonal OSC line confirmed that these categories reflect stable genomic insertions rather than population mosaicism (Additional file 1: Fig. S3D, E).

The three transposon groups largely mirror whether a transposon family is under piRNA control. Insertions of transposon families whose expression is strongly repressed by piRNAs in OSCs (e.g. *gypsy*, *mdg1*, *412*) were almost exclusively assigned to groups 1 and 2 (Fig. 3E-G). By contrast, those from families lacking antisense piRNAs (e.g. *copia*, *springer*) or transcriptional activity in OSCs (e.g. *roo*, *Doc*, *mdg3*) were largely placed in group 3 (Fig. 3E, F). These results reinforce a model where Piwi-dependent heterochromatin formation requires both the transcriptional activity of the transposon and the presence of cognate antisense piRNAs.

Unexpectedly, we identified striking intra-family exceptions to these rules. For example, a *copia* insertion within the *msn* gene formed one of the strongest Piwi-dependent heterochromatin domains in the genome (Fig. 3H, Additional file 1: Fig. S3C). This was surprising because *copia* is highly expressed In OSCs, lacks antisense piRNAs, and most of the other ~150 *copia* insertions are devoid of heterochromatin (Additional file 1: Fig. S3F). Closer inspection revealed that nearly all heterochromatin-marked *copia* insertions (we made similar observations for *springer*) reside in antisense orientation within the introns of actively transcribed host genes (Additional file 1: Fig. S3G). In this configuration, the abundant *sense* piRNAs produced by these elements (Fig. 3F) can recognize the *antisense* TE sequence within the nascent host pre-mRNA, thereby nucleating local heterochromatin domains (Fig. 3H).

Together, these results demonstrate how a precise transposon map, integrated with functional datasets, reveals the nuanced rules governing how the nuclear piRNA pathway controls gene expression and chromatin patterns.

### The OSC *flamenco* cluster and its impact on transposon piRNA profiles

Ovarian somatic cells, including the OSC line, predominantly generate transposon antisense piRNAs, with the genetically identified *flamenco* locus [43, 44] serving as the principal genomic source [39, 45, 46]. Located at the boundary between euchromatin and pericentromeric heterochromatin on chromosome X, *flamenco* exhibits high structural diversity across *Drosophila* strains [47]. Consistent with this, our comparison of the OSC assembly with the dm6 reference identified *flamenco* as one of the most divergent genomic regions (Additional file 1: Fig. S4A, B).

**Fig. 4:**
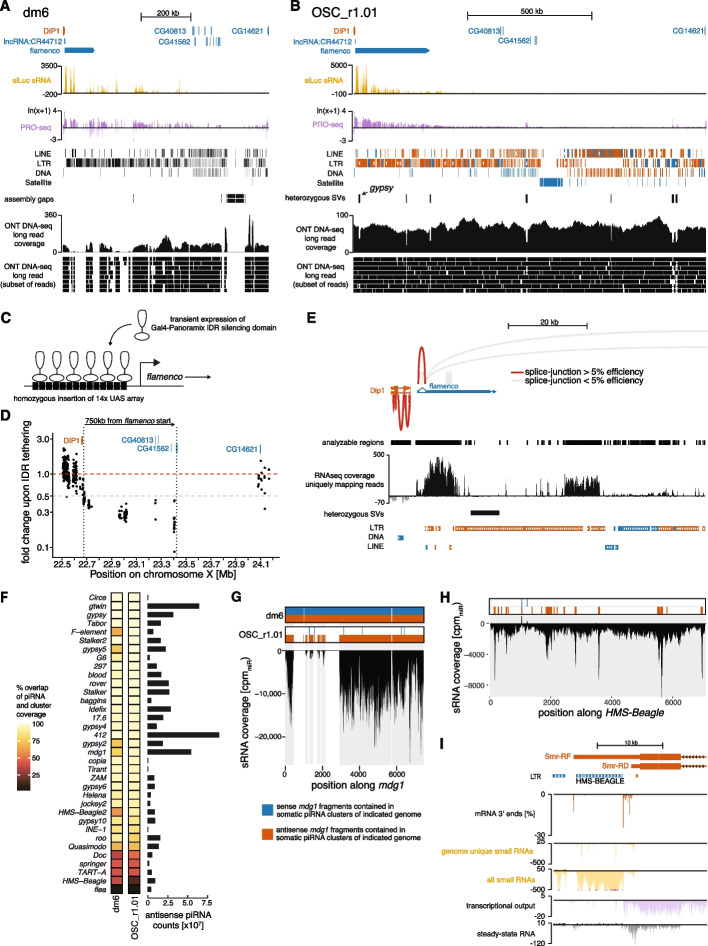
Reconstruction of the *flamenco* cluster in OSCs reveals its transcriptional extent and impact on transposon piRNA profiles. **A** Genome browser view of the extended *flamenco* locus (between *DIP1* and *CG14621*) in dm6, indicating gene annotations, transposon insertions, and assembly gaps. Small RNA data (genome-unique reads) were normalized to 1 million miRNAs. PRO-seq signal (genome-unique reads) represents ln(x+1)-transformed coverage per million (negative counts indicate genomic minus strand reads). At the bottom, OSC-derived ONT DNA-seq data are shown as coverage and representative reads. The 'assembly gaps' track indicates the positions of gaps in the dm6 reference genome assembly. **B** Genome browser view of the extended *flamenco* locus (between *DIP1* and *CG14621*) in OSC_r1.01, indicating gene annotations, transposon insertions (antisense in orange, sense in blue), and no assembly gaps. Small RNA and PRO-seq tracks as in (A). At the bottom, OSC-derived ONT DNA-seq data are shown as coverage and representative reads. Heterozygous structural variants from ONT reads are indicated, including a labeled heterozygous *gypsy* insertion. **C** Schematic of the *flamenco* silencing experiment. **D** 1-kb tile analysis of fold change in small RNA coverage (genome-unique reads) upon *flamenco* silencing across the extended *flamenco* locus in OSC_r1.01. Dotted lines mark the region from the *flamenco* TSS to the most distal analyzable point showing silencing. **E** Genome browser view of the initial 73 kb of *flamenco* in OSC_r1.01, including *DIP1*. All identified splice junctions are shown. Analyzable (genome-unique) regions are indicated; multi-mapping regions were excluded from splice assessment. RNA-seq coverage used for splice analysis is shown as counts per 10 million genome-uniquely mapped reads. **F** Heatmap showing the concordance (percent overlap) between transposon antisense piRNA coverage and their corresponding fragments within the *flamenco*, *20A*, and *77B* piRNA clusters, separately for dm6 (left) and OSC_r1.01 (right). Shown to the right are the antisense piRNAs targeting each transposon (normalized to 1 million miRNAs). **G** Histogram of piRNA coverage along *mdg1* (negative read-counts indicate coverage on the antisense strand). Above, *mdg1* fragments present in *flamenco*, *20A*, and *77B* (dm6 and OSC_r1.01) are shown (sense: blue; antisense: orange). Regions with antisense piRNA coverage are shaded gray. **H** Histogram of piRNA coverage along *HMS-Beagle* (negative read-counts indicate coverage on the antisense strand). Above, *HMS-Beagle* fragments present in *flamenco*, *20A*, and *77B* in OSC_r1.01 are shown (sense: blue; antisense: orange). Regions with antisense piRNA coverage are shaded gray. **I** Genome browser view of the antisense *HMS-Beagle* insertion in the *Smr* 3′ UTR. Individual genic 3′ ends from ONT direct RNA-seq 3′-end quantification are shown as percent of all detected *Smr* 3′ ends. RNA-seq and PRO-seq are shown as counts per million; small RNAs are shown as genome-unique and all-mapping reads (normalized to 1 million miRNAs; negative read-counts indicate coverage on the antisense strand)

Alignment of Oxford Nanopore (ONT) genomic reads from OSC DNA to the different assemblies underscored these differences. When mapped to dm6, ONT reads aligned only in fragmented segments with poor coverage (Fig. 4A). By contrast, mapping the same reads to OSC_r1.01 produced seamless, continuous alignments with uniform coverage, interrupted only by seven heterozygous structural variants, including a heterozygous *gypsy* insertion at the *flamenco* 5′ end (Fig. 4B). Unlike the dm6 reference, which contains an assembly gap within *flamenco* (Fig. 4A), OSC_r1.01 provides a contiguous, gapless reconstruction of the entire extended OSC *flamenco* region flanked by *DIP1* and *CG14621* (Fig. 4B). Based on this assembly, the extended *flamenco* locus spans approximately 1.5 Mb, raising the question of how far functional transcription and piRNA production extend beyond the annotated promoter near *DIP1*.

To address this, we inserted UAS sites upstream of the *flamenco* promoter on both alleles of the diploid OSC line (Fig. 4C). Transient transfection with a fusion protein between the GAL4 DNA-binding domain and the transcriptional repressor domain of Panoramix [29] suppressed piRNA production from *flamenco* (Fig. 4D) without affecting piRNAs derived from *cluster 20A*, *cluster 77B*, or genic 3′ UTRs (Additional file 1: Fig. S4C-G). At *flamenco*, piRNA loss extended at least as far as ~730 kb downstream of the promoter, demonstrating that transcription across much of this expanded locus contributes to piRNA generation (Fig. 4D). To determine the impact of *flamenco* silencing on global piRNA populations, we quantified changes in antisense piRNA levels for transposons represented within the OSC *flamenco* locus. Most families with antisense insertions in *flamenco* showed a strong depletion of antisense piRNAs upon repression, confirming *flamenco* as their primary source (Additional file 1: Fig. S4H). Interestingly, while *roo* also has antisense fragments within *flamenco*, its antisense piRNA levels increased upon *flamenco* silencing. We speculate that a *roo* antisense transcript from an uncharacterized locus is derepressed in *flamenco*-depleted cells, leading to the observed piRNA increase.

Analysis of uniquely mapping small RNAs revealed that piRNA output diminishes gradually along *flamenco*, dropping below 5% of peak levels beyond 500 kb from the transcription start site (Fig. 4B). While *flamenco* transcripts have been proposed to undergo extensive splicing [46], our analysis of ribo-zero RNA-seq data provided no evidence for additional splice events beyond the previously described intron near the promoter (Fig. 4E, Additional file 1: Fig. S4I). These findings suggest that the decline in piRNA output reflects a decay in transcriptional activity rather than alternative processing, a conclusion supported by PRO-seq data (Fig. 4B).

Finally, we asked to what extent the sequence content of the three active piRNA clusters in OSCs—*flamenco*, *20A*, and *77B*—defines the cellular transposon piRNA repertoire. We compared OSC piRNA profiles to the exact antisense transposon content within these three clusters using both dm6 and OSC_r1.01. The OSC_r1.01 assembly markedly improved the correlation between cluster content and observed piRNA profiles (Fig. 4F). For example, the LTR retrotransposon *mdg1* generates an exclusively antisense piRNA profile with several pronounced gaps in OSCs (Fig. 4G). The dm6 version of *flamenco* contains complete sense and antisense *mdg1* copies, which is inconsistent with the observed data. In contrast, OSC_r1.01 contains only *mdg1* antisense fragments in *flamenco* that correspond precisely to regions of abundant piRNA production (Fig. 4G). Notably, the low-abundance piRNAs mapping to *mdg1* (nt 1,000–2,100) precisely matched antisense fragments uniquely found in cluster *20A*, which is transcribed at lower levels than *flamenco*.

Overall, the sequence content of these three clusters accounts for the vast majority of transposon antisense piRNAs in OSCs. Rare exceptions, such as piRNAs targeting *flea* and *HMS-Beagle*, likely originate from other sources (Fig. 4H). For instance, an antisense *HMS-Beagle* insertion within the 3′ UTR of *Smr* likely accounts for the observed antisense piRNAs (see also [48]), while more prominent peaks are explained by small *HMS-Beagle* fragments in *flamenco* (Fig. 4I). In summary, the OSC_r1.01 assembly provides a complete reconstruction of the *flamenco* cluster, reveals its 700-kb transcriptional extent, and demonstrates how its specific sequence architecture dictates the somatic piRNA repertoire.

## Conclusions

We present a high-quality de novo assembly of the *Drosophila* ovarian somatic cell (OSC) genome, providing a reference for one of the most widely used cell systems in piRNA research. This resource closes an important gap between the biology of OSCs and reliance on the *Drosophila melanogaster* dm6 reference genome, which differs considerably in transposon content and piRNA cluster composition.

The OSC genome resolves the structure and transcriptional extent of the *flamenco* piRNA cluster, demonstrates how cluster sequence content directly determines the piRNA repertoire targeting most transposons in this cell line, and clarifies how orientation and genomic context of transposon insertions dictate whether they are targeted by the nuclear Piwi pathway. These findings showcase the value of accurate genome assemblies for interpreting small RNA populations and their functional consequences.

One of the goals of this work was to establish the OSC genome assembly as a useful community platform. By providing comprehensive gene and transposon annotations together with an interactive UCSC genome browser hub, it enables seamless integration of diverse datasets. We anticipate that this accessibility will aid the design of targeted experiments, facilitate cross-study comparisons, and accelerate discovery in piRNA biology. We note that Siomi, Iwasaki and colleagues recently published a genome assembly of the OSC line used in their laboratory [40], which differs slightly from the line sequenced by us [33]. As their assembly lacks annotations and a browser-compatible resource, we hope that a common platform featuring both genome assemblies will be established in the future.

## Materials & methods

All materials used in this study, including cell lines and commercial kits with their respective order numbers, are detailed in the Key Resources Table (Additional file 1: Table S3).

### OSC cell culture

Ovarian somatic cells (OSCs) were received as a gift from the Siomi laboratory in 2010 and we established a master cell-bank from these original cells. OSCs used for this study have been derived from this cell-bank. Cell line identity was confirmed via origin and the consistent maintenance of cell-type-specific functional properties (piRNA pathway activity and characteristic morphology); no formal authentication or mycoplasma testing was performed for this study.

OSCs were maintained at 27 °C in a humidified incubator with 2.5% CO₂, as previously described [12, 13]. Cells were cultured in M3 basal medium supplemented with 10% fetal bovine serum (FBS), insulin, glutathione, and fly extract. For siRNA-mediated knockdown, OSCs were electroporated using either an Amaxa 2B nucleofector (Lonza) with Kit V and program T-029, or an Amaxa 4D nucleofector with Buffer SF and program DG150. A total of 4 × 10⁶ cells were transfected in 100 µL electroporation buffer supplemented with 4 µL siRNA (50 µM stock). A second transfection was performed 48 h after the initial transfection, and cells were harvested 96 h post the initial transfection for downstream analyses.

### Genomic DNA isolation

Genomic DNA was isolated from OSC cells using a modified protocol based on Jain et.al [49]. OSCs from T75 flasks were washed with (Phosphate Buffered Saline) (NaCl 8g/L; KCl 0.2 g/L; Na_2_HPO_4_ 1.15 g/L; KH_2_PO_4_ 0.2 g/L) and lysed directly in 10 mL lysis buffer (200 mM NaCl, 100 mM Tris-HCl pH 8.5, 50 mM EDTA, 0.5% SDS, 50 µL Qiagen Puregene Proteinase K per 10 mL). The lysate was incubated at 60 °C for 5 h with occasional gentle mixing. After cooling, 100 µL Qiagen RNase A solution was added and incubated for 2 h at 37 °C. Following RNase treatment, the lysate was transferred to a Qiagen MaXtract high-density tube for sequential extractions. It was first mixed with 10 mL phenol (pH 8.0) by rotation (20 min, 20 rpm), then centrifuged (10 min, 1,500 g, 4 °C). The aqueous phase was transferred to a new MaXtract tube, mixed with 5 mL phenol (pH 8.0) and 5 mL chloroform, and rotated (10 min, 20 rpm) before a second centrifugation. The final aqueous phase was mixed with 7 mL isopropanol to precipitate DNA, which was then spooled with a sealed Pasteur pipette. The DNA was washed three times with 70% ethanol and resuspended in 100 µL Tris-HCl (pH 8.7) by overnight rotation at 4 °C for solubilization.

### Nanopore DNA library preparation and sequencing

Libraries were prepared using either the LSK108 ligation sequencing kit (Oxford Nanopore Technologies) or the RAD002 rapid sequencing kit. LSK108 libraries were constructed according to the manufacturer’s standard protocol to ensure efficient adaptor ligation and clean-up. RAD002 libraries followed the modified protocol of Jain et al. [49]. In this approach, the amount of transposase used during the fragmentation step was reduced to minimize over-fragmentation and preserve long DNA fragments, while the incubation time with the adaptor-ligation mix was extended to improve adaptor attachment efficiency. These modifications enabled the recovery of ultra-long DNA molecules (>100 kb). To further protect high–molecular weight DNA during handling, library clean-up conditions were optimized to reduce mechanical shearing, and all pipetting steps were performed with wide-bore tips using slow, minimal mixing. Sequencing libraries were directly loaded onto R9.4 (FLO-MIN106) flow cells and ran on the MinION device, following the manufacturer’s guidelines for flow cell priming, sample loading, and temperature control. Sequencing performance was monitored in real time with MinKNOW, and runs were continued until active pore counts declined substantially.

### Nanopore RNA library preparation and sequencing

Total RNA was isolated from 90% confluent T75 flasks of OSC cells. Cells were washed three times with PBS, lysed with 10 ml of Trizol, and incubated on a shaker for 30–60 minutes. The lysate was transferred to a 15 ml Falcon tube containing phase lock gel (2g transferred from 50 ml Maxtract tubes per 15 ml Falcon), mixed with 2 ml of chloroform by careful inversion, and centrifuged at 4800 g for 10 minutes at 20 °C using a swing-out rotor. The aqueous phase was transferred to a fresh Falcon tube, 7 ml of isopropanol was added, and the mixture was incubated at room temperature for 15 minutes. RNA was then pelleted by centrifugation at 20,000 g for 1 hour at 4 °C in a fixed-angle rotor. The supernatant was removed, and the pellet was washed with 75% ethanol. After complete removal of ethanol, the RNA pellet was resuspended in 500 µL of nuclease-free water without pipetting for mixing and carefully transferred to an Eppendorf tube. RNA concentration was measured using Qubit RNA BR.

mRNA was selected using oligo(dT) Dynabeads. A 200 µL aliquot of Dynabead slurry was washed twice with 2x binding buffer (10 mM Tris-HCl pH 7.5, 1.0 M LiCl, and 2mM EDTA) and resuspended in 100 µL of 2x binding buffer. Total RNA was heat-denatured at 65 °C for 5 minutes and snap-cooled on ice. Washed beads were added to the heat-denatured RNA, and the mixture was incubated with overhead rotation for 15 minutes. After incubation, the supernatant was removed, and the beads were washed three times with 500 µL of wash buffer (10 mM Tris-HCl pH 7.5, 0.15 M LiCl, and 1mM EDTA), resuspending the beads each time. mRNA was eluted into 12 µL of nuclease-free water by incubation at 80 °C for 1 minute. mRNA concentration was measured using Qubit RNA BR.

Nanopore direct RNA sequencing libraries were prepared from 10 µL of eluted mRNA following the RNA002 protocol (Oxford Nanopore Technologies). Half of the prepared library was used on a fresh flow cell for sequencing.

### Illumina DNA library preparation

Genomic DNA was fragmented using a Covaris E220 instrument with microTUBEs containing AFA fibers, following the manufacturer’s settings for a 400 bp peak size (Peak Incident Power = 140, Duty Factor = 10%, Cycles per Burst = 200, Treatment Time = 55 s). Size selection was performed with AMPure XP beads (Beckman Coulter). Long fragments were first removed by adding 53 µL of bead slurry per 100 µL of genomic DNA. Subsequently, an additional 25 µL of AMPure XP beads was added, and DNA fragments were purified according to the manufacturer’s protocol. Libraries were prepared using the NEBNext Ultra DNA Library Prep Kit (New England Biolabs) according to the manufacturer’s instructions, except for the final amplification step. For amplification, libraries were PCR-amplified using KAPA HiFi polymerase with EvaGreen fluorescence incorporated for real-time monitoring. PCR reactions were terminated once 3500-4000 RFUs (relative fluorescent units as measured by the BioRad CFX Connect Real Time PCR Detection System without baseline correction) were reached to prevent over-amplification. After purifications using the Zymo DCC5 columns, samples were sequenced (PE100, Illumina HiSeq2000).

### Hi-C library preparation

Hi-C libraries were generated as previously described [50], with minor modifications. OSCs were harvested, washed in PBS, and resuspended in M3 medium containing 1% formaldehyde at a density of 5 × 10⁶ cells/mL. After incubation for 10 minutes at room temperature (RT) with gentle rotation, 2 M Tris-HCl (pH 8.0) was added to a final concentration of 0.125 M to quench crosslinking. Samples were incubated for 5 minutes at RT, followed by 20 minutes on ice. Cells were pelleted, washed twice with PBS, and stored in aliquots of 5 × 10⁶ cells at –80 °C. Two OSC aliquots (5 × 10⁶ cells each; 1 × 10⁷ total) were processed in parallel. Cells were resuspended in 1 mL ice-cold lysis buffer, incubated for 30 minutes at 4 °C with rotation, and pelleted (2,500 g, 5 minutes, 4 °C). Pelleted nuclei were resuspended in 1 mL lysis buffer (10 mM Tris ph8; 10 mM NaCl; 0.2% Igepal CA-630; Protease inhibitor), pelleted again, and finally resuspended in 400 µL 1.2× NEB buffer 3.1, minimizing additional wash steps to reduce clumping.

Nuclei were digested overnight at 37 °C with rotation (800 rpm) using 30 U DpnII (NEB) in a final volume of 80 µL. After digestion, pellets were resuspended in 52 µL fill-in mix containing 2.2 µL biotin-14-dATP (1 mM, Jena Bioscience), 1.1 µL dNTP mix (2 mM dCTP, dGTP, dTTP each), 11 µL Klenow fragment, 5.72 µL NEB buffer 2, and nuclease-free water to volume, and incubated for 1.5 h at 37 °C with rotation. Filled-in nuclei were resuspended in a 250 µL ligation mix (27.5 µL Thermo T4 DNA ligase buffer, 2.2 µL 10% Triton X-100, 2.25 µL BSA [10 mg/mL], 232.5 µL H₂O, and 11 µL T4 DNA ligase). Samples were incubated at RT for 5 h with rotation. Aliquots (10 µL) were collected at digestion and ligation steps as process controls. Post-ligation, reactions were diluted to 500 µL with PBS, supplemented with NaCl (500 mM final concentration) and 10 µL proteinase K (20 mg/mL), and incubated overnight at 65 °C. DNA was purified by two rounds of phenol–chloroform extraction, followed by a chloroform wash, and ethanol precipitation in the presence of glycogen. Pellets were resuspended in 150 µL H₂O (controls in 10 µL), treated with RNase A (2 µL [10 U/µL], 30 minutes, 37 °C), and quantified using Qubit DNA BR. 130 microliters of DNA sample were transferred to Covaris microTUBEs and sheared on a Covaris E220 (<10 °C, intensifier inserted, 105 W peak power, 5% duty factor, 200 cycles/burst, 55 s). DNA was rinsed from Covaris tubes with 80 µL EB and subjected to two-step Ampure XP bead size selection (0.44× and 0.18×). DNA was eluted in 100 µL H₂O. Biotinylated DNA was captured with Dynabeads MyOne Streptavidin C1, pre-washed in Tween wash buffer (5 mM Tris-HCl pH 7.5, 0.5 mM EDTA, 1 M NaCl, 0.05% Tween-20). Beads were resuspended in 100 µL 2× binding buffer (10 mM Tris-HCl pH 7.5, 1 mM EDTA, 2 M NaCl), mixed with DNA, and incubated for 1.5 h at room temperature with rotation. Beads were washed twice in 500 µL Tween wash buffer, twice in H₂O, and resuspended in 50 µL H₂O. Bead-bound DNA was subjected to end repair (7 µL End Prep buffer, 3 µL enzyme mix; 30 minutes at 20 °C then 30 minutes at 65 °C), adaptor ligation (2.5 µL NEB Illumina adaptor, 1 µL ligation enhancer, 30 µL ligation master mix; 15 minutes at 20 °C), and USER enzyme (NEB) treatment (3 µL; 15 minutes, 37 °C) using the NEBNext Ultra IIDNA Library Prep Kit. Beads were washed four times with 500µL Tween wash buffer. DNA was eluted in 20 µL pre-warmed elution buffer (10 mM EDTA, 95% formamide, 65 °C), ethanol-precipitated with glycogen and NaCl, and resuspended in 15 µL H₂O. PCR amplification was performed with KAPA HiFi polymerase (25 µL master mix, 2 µL EvaGreen, 2.5 µL 10 µM barcoded primer, 1 µL 25 µM universal forward primer, 4.5 µL water). Cycling proceeded for 5 cycles, stopping when real-time fluorescence monitoring reached 3,500-4,000 RFU (relative fluorescent units as measured by the BioRad CFX Connect Real Time PCR Detection System without baseline correction). PCR products were diluted, purified using Zymo DNA Clean & Concentrator-5, and eluted in 15 µL H₂O. Final libraries were quantified with Qubit, quality-checked on a Fragment Analyzer, and sequenced (PE75, illumina NextSeq 550).

### sRNA-seq library preparation

sRNA-seq libraries were prepared by first purifying Argonaute–sRNA complexes with homemade TraPR resin columns as described in [51]. OSCs were harvested using trypsin, washed 2x with PBS and stored at −70°C after snap freezing cell-pellets in liquid nitrogen. OSCs were lysed by adding 330 µL of TraPR lysis buffer. OSC lysates were clarified, normalized to equal protein amounts (depending on the experiment 70–120 µg total protein) after Bradford measurements, applied to TraPR columns, and Argonaute-bound RNAs were eluted, followed by phenol–chloroform extraction and precipitation. This column-based isolation bypasses conventional gel-based size selection and provides highly specific enrichment for Ago-associated small RNAs. To minimize ligation bias and facilitate library multiplexing, adaptors were designed with random nucleotides placed adjacent to the ligating end [52]. The 3′ adaptor carried six random bases at its 5′ terminus and contained sample barcodes (X) for multiplexing (/5rApp/NN NNN NXX XXX AGA TCG GAA GAG CAC ACG TCT/3ddC/). The 5′ adaptor was an RNA oligo bearing four random nucleotides at its 3′ terminus. After 3′ adaptor ligation with T4 RNA ligase 2, truncated KQ (NEB), samples were pooled as necessary, spiked with fluorescently labeled and ligation-blocked oligos and gel-purified on a 12.5% polyacrylamide–urea gel. After imaging using a Licor Odyssey gel-regions flanked by the fluorescent oligos were excised. Small RNAs were isolated using the Zymo ZR small RNA PAGE Recovery kit and subjected to 5′ adaptor ligation with T4 RNA ligase 1. Following cleanup with Zymo RCC5 columns, reverse transcription using SuperScript II was performed. Libraries were PCR-amplified with KAPA HiFi polymerase and dual-index barcoded primers (NEB). Amplification was monitored in real time with EvaGreen, and reactions were stopped upon reaching 3,500–4,000 RFU (relative fluorescent units as measured by the BioRad CFX Connect Real Time PCR Detection System without baseline correction) to prevent overamplification. Final libraries were size-selected on 2.5% low-melt agarose gels, gel-purified (Zymo), quantified with Qubit, multiplexed and sequenced (SE50, Illumina NovaSeq6000).

### PROseq library preparation

PROseq libraries were prepared using a protocol modified from Mahat et al. [53] from both isolated nuclei and permeabilized cells. Cells were harvested, washed with ice-cold PBS, and counted. Nuclei were isolated by resuspension in douncing buffer (10 mM Tris-HCl pH 7.4, 300 mM Sucrose, 3 mM CaCl_2_, 2 mM MgCl_2_, 0.1% Triton X-100, supplemented with 0.5 mM DTT, protease and RNase inhibitors), dounced 25 times with a tight pestle, and pelleted. Permeabilized cells were prepared by resuspension in permeabilization buffer (10 mM Tris-HCl pH 7.4, 300 mM Sucrose, 10 mM KCl, 5 mM MgCl₂, 1 mM EGTA, 0.05% Tween, 0.1% NP40 substitute, supplemented with 0.5 mM DTT, protease, and RNase inhibitors), incubated on ice for 5 minutes, and pelleted. Both nuclei and permeabilized cells were resuspended in storage buffer (10 mM Tris-HCl pH 8.0, 25% glycerol, 5 mM MgCl_2_, 0.1 mM EDTA, supplemented with 5 mM DTT), aliquoted, and snap-frozen in liquid nitrogen. Nuclear run-on reactions were performed using 2xNRO buffer supplemented with biotin-11-CTP and biotin-11-UTP (for 2-biotin mixes) or single biotinylated nucleotides (for 1-biotin mixes), ATP, GTP, and RNaseOUT. Samples were incubated at 30 °C for 3 minutes, followed by RNA extraction using Trizol-LS and chloroform. RNA was precipitated with Glycoblue and 100% ethanol, incubated for 15 minutes at room temperature, and pelleted by centrifugation (16,000 x G, 1 hour, 4 °C). Pellets were washed with 75% ethanol and stored at −80°C. Isolated RNA was fragmented by heat denaturation (65°C for 40 seconds) and alkaline hydrolysis (1N NaOH for 10 minutes on ice), then purified using P30 columns. Biotinylated RNA was enriched using MyOne C1 streptavidin beads, washed with high-salt, PROseq binding, and low-salt buffers, and eluted with Trizol and chloroform extraction. RNA was precipitated with Glycoblue and 100% ethanol. After decapping and hydroxyl repair using TAP and PNK enzymes followed by phenol purification, libraries were generated under sRNA-seq ligation conditions. 3′ adaptor ligation was performed with T4 RNA ligase 2, truncated KQ (NEB), using adaptors containing four random nucleotides at the 5′ end [52]. After a second streptavidin purification, 5′ adaptor ligation was performed with T4 RNA ligase, using adaptors containing four random nucleotides at the 3′ end, followed by a third streptavidin purification. Reverse transcription was carried out with SuperScript II using an adaptor-specific primer. Libraries were PCR-amplified with KAPA HiFi polymerase and barcoded Illumina primers in reactions monitored by EvaGreen real-time fluorescence. Reactions were stopped upon reaching 3,500–4,000 RFU (relative fluorescent units as measured by the BioRad CFX Connect Real Time PCR Detection System without baseline correction) to prevent overamplification. PCR products were separated on 2.5% low-melt agarose gels, gel-purified (Zymo), and multiplexed for sequencing (PE50, Illumina HiSeq2500).

### RNAseq library preparation

Total RNA was isolated using Trizol (Invitrogen) according to the manufacturer’s instructions. Ribosomal RNA was depleted using an RNAseH based protocol [54, 55]. In brief, standard 50-mer DNA oligos complementary to rRNA precursor [56] and mitochondrial rRNA sequences were annealed to 1 µg total RNA in Hybridase buffer with EDTA. Samples were denatured (95 °C, 3 min), cooled gradually to 45 °C, and incubated with Thermostable Hybridase RNAseH (1 h, 45 °C) to specifically degrade rRNA. Following digestion, samples were treated with Turbo DNAse (30 min, 37 °C) and purified using RNA Clean & Concentrator-5 columns (Zymo). Elution was carried out in NEBNext Ultra II First Strand Synthesis buffer for downstream library preparation. RNA-seq libraries were generated using the NEBNext Ultra II Directional RNA Library Prep Kit (New England Biolabs) according to the manufacturer’s instructions, except for the final amplification. Libraries were PCR-amplified with KAPA HiFi polymerase and barcoded Illumina primers, with amplification monitored in real time using EvaGreen fluorescence. Reactions were stopped upon reaching 3,500–4,000 RFU (relative fluorescent units as measured by the BioRad CFX Connect Real Time PCR Detection System without baseline correction) to prevent overamplification. Libraries were then purified, quantified by Qubit, assessed for fragment size, and multiplexed for Illumina sequencing.

### Generation of UAS landing-site cell lines

A donor repair template was generated by cloning ~1 kb homology arms flanking an attP-flanked Puro-mCherry cassette into a plasmid backbone. The construct was assembled by Gibson assembly, propagated in *E. coli* STBL3, and sequence-verified by Sanger sequencing (Plasmid map available at https://github.com/BrenneckeLab/Handler_2026-OSC-genome).

A sgRNA targeting the chosen insertion site within the *flamenco* upstream region (target: TATAAAAGTTACAAAATACG) was designed using CHOPCHOP, synthesized as complementary oligonucleotides, and cloned into a Cas9 expression vector (Addgene 49330 [57]). OSCs were co-transfected with the landing-site donor and sgRNA/Cas9 plasmids using the Amaxa 4D nucleofection system (buffer SF, program DG150). Forty-eight hours post-transfection, cells were replated into 10-cm dishes at varying densities (2.5%, 5%, 10% of total cells). Selection was initiated with puromycin (1:1,500) five days after plating. Resistant colonies were picked after 7 days, expanded in 96-well plates, and subsequently scaled up. Clonal integration was assessed by long-range PCR spanning the integration site and ddPCR using primers specific for the donor cassette and endogenous controls. The assays confirmed the landing site integrity, from which a verified homozygous clone was selected.

### *flamenco* silencing

A plasmid containing an attB–selection cassette–14xUAS–attB was constructed. The selection cassette encoded *mCherry–Blasticidin*, driven by the *D. yakuba tj* enhancer with the *armi* 3′ UTR (Plasmid map available at https://github.com/BrenneckeLab/Handler_2026-OSC-genome). Homozygous *flamenco* landing site (LS) OSCs were co-transfected with the UAS construct and a φC31 integrase expression plasmid (Addgene #26290) using Amaxa 4D nucleofection (buffer SF, program DG150). Five days post-transfection, feeder OSCs were prepared by diluting OSCs to 1 × 10^6^ cells/mL, irradiating twice for 20minutes (dose of ~6Gy each irradiation) with mixing between treatments, and plating 1 × 10^5^ feeder cells per well of a 96-well plate. The following day, single cells that were GFP positive and mCherry negative were sorted into feeder-containing wells. After 14 days, wells with colonies were identified by scanning the plates for GFP signal (Sapphire - Azure Biosystems). Identified clones were transferred to a new plate and expanded. Genomic PCR was used to assess cassette integration, and Sanger sequencing confirmed integration orientation. Clones homozygous for UAS insertions oriented towards *flamenco* were subsequently transfected with tethering plasmids encoding either Gal4 alone or Gal4 fused to the Panoramix IDR silencing domain [29]. Transfections were repeated after 48 h to sustain tethering. At 6 and 7 days post transfection, cells were harvested by FACS, gated on reduced GFP reporter signal from the co-silenced selection cassette. For Gal4-only controls, no FACS enrichment was applied. Small RNAs were isolated from sorted cells and sequenced as described in the small RNA sequencing section, except that the 3′ adaptor lacked a barcode and carried only four random nucleotides at its 5′ end.

#### Computational analyses

The computational results presented were obtained using the CLIP cluster (https://clip.science) utilizing standard tools for data-analysis and quality control. Statistical analyses were performed in R (v4.3.2). Data visualization was performed using ggplot2 and associated packages. A comprehensive list of all software tools with version numbers and citations is provided in Additional file 1: Table S4. All code created for this publication are available at https://github.com/BrenneckeLab/Handler_2026-OSC-genome

### Nanopore DNA sequencing base-calling and read processing

Raw multi-read FAST5 files were base-called on GPUs with ONT Guppy using the super-accuracy model dna_r9.4.1_450bps_sup (Oxford Nanopore Technologies). Base-calling was performed without quality filtering to retain all reads for downstream QC and filtering (--disable_qscore_filtering). Post-base-calling, FASTQ files from each batch (including pass and fail subdirectories) were concatenated and processed in parallel to generate adaptor-trimmed and untrimmed read sets. Adaptor trimming was performed with Porechop [58] using default settings with minimum split read size of 1,000 nt and adaptor detection on a 1,000-read subsample (--min_split_read_size 1000 --check_reads 1000). For the untrimmed set, chimeric reads were discarded (--discard_middle) but end adaptors were retained. Read length and average per-base quality scores were computed from FASTQ quality strings using a precomputed Phred score lookup table, and appended to read headers (LENGTH=, avgQUAL=) with seqkit [59] and mawk.

### Nanopore direct RNA sequencing base-calling and read processing

Direct RNA multi-read FAST5 files were base-called on GPUs with ONT Guppy using the appropriate direct RNA configuration for the flow cell/kit (Oxford Nanopore Technologies). Base calling emitted all reads (--disable_qscore_filtering) while preserving raw signal and disabling base-caller trimming where applicable (--fast5_out --trim_strategy none). Post-base-calling, per-batch FASTQ files were concatenated and processed to annotate read bodies upstream of the poly(A) tail and to estimate poly(A) lengths. Base-called FAST5 files were converted to gzip-compressed FAST5 (compress_fast5, ONT) and poly(A) tails were identified with tailfindr in R, yielding per-read tail metrics (tail_end_base_index, tail_length) [60]. For each read, sequence and quality strings were truncated at tail_end_base_index to retain the transcribed body upstream of the poly(A) tract, and metadata were appended to read headers (LENGTH=, avgQUAL=, polyAlength=) with seqkit [59] and mawk. Reads without a detectable tail were flagged (polyAlength=notDetected) and listed for QC. For 3′ UTR annotation, only direct RNA reads with a detected poly(A) tail were used.

### Genome assembly and haplotig purging

Nanopore reads were filtered to retain sequences >15,000 nt, scrubbed with Yacrd (-c 4 -n 0.4 scrubb) [61], and quality-trimmed with Filtlong (--target_bases 14400000000; https://github.com/rrwick/Filtlong) to obtain approximately 100× coverage. Filtered reads were assembled with Shasta using custom settings (see associated code deposition) [62]. Redundant haplotigs were identified and removed with Purge Haplotigs [63] (modified version https://gitlab.com/dominik-handler/purgehaplotigs). Read-depth histograms were generated by mapping reads with Minimap2 [64] and processed with purge_haplotigs hist. Repeat-masked regions were annotated with RepeatMasker (Smit, AFA, Hubley, R & Green, P. *RepeatMasker Open-4.0*.2013-2015 http://www.repeatmasker.org) (NCBI/RMBLAST v2.10.0+, CONS-Dfam_3.1-rb2018102 [36]). Haplotig purging was performed using purge_haplotigs cov -l 10 -m 48 -h 180, followed by purging with repeat annotations taken into account. Overlapping contig ends were trimmed with purge_haplotigs clip using default settings. Mis-assembly correction was performed based on read-alignment of Oxford Nanopore Technologies (ONT) reads and analysis of read-coverage gaps. ONT long reads were aligned to the initial assembly using minimap2 (map-ont mode) with secondary alignments excluded. Alignments were filtered to remove unmapped and supplementary reads, converted to BED format using samtools [65] and bedtools [66], and sorted by genomic coordinates. Coverage gaps indicative of potential mis-assemblies were identified using bedtools genomecov to generate per-base coverage across all contigs. Regions with coverage below 10× and located more than 30 kb from contig ends were flagged as candidate mis-assembly sites, provided the gap spanned at least 20 bp. Adjacent gaps within 1 kb were merged using bedtools merge to define consolidated break regions. For contigs containing identified gaps, splitting coordinates were determined by creating intervals between consecutive gap regions, with each resulting fragment assigned a unique identifier. Contigs without detected mis-assemblies were retained intact with their original identifiers. The final split coordinates were used with bedtools getfasta to extract individual contig fragments, generating a corrected assembly file for subsequent Hi-C scaffolding. After mis-assembly correction, scaffolding was performed using Hi-C contact data. Raw Hi-C read pairs were aligned to the split genome assembly with BWA-MEM [67] using parameters optimized for Hi-C data (-A 1 -B 4 -E 50 -L 0). Alignments were filtered to remove low-quality reads and artifacts with the filter_five_end.pl script provided in the SALSA2 package [68, 69] then combined and sorted [68, 69]. Read groups were assigned with Picard AddOrReplaceReadGroups (“Picard Toolkit.” 2019. Broad Institute, GitHub Repository. https://broadinstitute.github.io/picard/; Broad Institute), and PCR duplicates were identified and removed using Picard MarkDuplicates. Scaffolding was then carried out with SALSA2, specifying GATC as the restriction enzyme motif to match the DpnII digestion used during Hi-C library preparation. Additional super-scaffolding was performed against the *Drosophila melanogaster* dm6 reference genome using RagTag (v2.1.0) [70] with default parameters. Gap-filling was then carried out in two successive rounds using an in-house pipeline supported by Oxford Nanopore long reads. Assembly gaps were first identified and flanking regions (20 kb on each side) extracted. Long reads were mapped to the flanks with minimap2 (map-ont mode), and candidate gap-spanning reads were selected. When a single bridging read was available, it was polished with racon to generate a gap-filling contig. In the absence of bridging reads, flanking reads were assembled de novo using wtdbg2/WTPOA [71] to produce contigs spanning both flanks. Candidate contigs were validated by realignment of the flanking regions and, if consistent, used to replace the corresponding gap sequence.

Heterozygous transposable element (TE) insertions that were lost during haplotig purging were recovered and reintegrated into the primary assembly using a custom computational pipeline. Nanopore reads ≥10 kb were first extracted using seqkit [59] and aligned to the reference assembly using minimap2 [64] with Oxford Nanopore Technologies (ONT) preset parameters (map-ont). Alignments were sorted using samtools [65], and structural variants were identified using Sniffles v1.012a [72] with a minimum variant length of 100 bp and no limit on the number of reported variants per locus. Structural variants were phased based on supporting read overlap: consecutive variants supported by reads overlapping by at least 25% with the previous variant's supporting reads were assigned to a new phase block. This approach allowed local distinction between variants on different haplotypes without requiring trio data or population-level information. To identify TE-containing insertions, sequences from insertion variants >50 bp were extracted and annotated using RepeatMasker with a custom TE consensus library based on RepBase [73] (available at https://github.com/BrenneckeLab/Handler_2026-OSC-genome). Insertions were retained for further processing only if >50% of their sequence was classified as TE-derived. TE insertions within 2 kb of each other and belonging to the same phase block (both for all structural variants and TE-specific phasing) were merged to consolidate fragmented calls. For each candidate TE insertion locus, reads supporting the variant were extracted and locally assembled using wtdbg2 [71] with parameters optimized for Nanopore data (genome size 30 kb, minimum read length 5,000 bp, with realignment and self-correction enabled, minimum alignment length 1,024 bp). Assembled contigs were polished using racon [74] with a match score of 8, mismatch penalty of −6, gap penalty of −8, and window size of 500 bp. To validate TE insertions for integration, the insertion sequence reported by Sniffles v1.012a was mapped to the assembled contig using minimap2. Genomic flanking sequences (10 kb upstream and downstream of the insertion site) were extracted from the reference assembly and mapped to the assembled contig. Insertions were validated when: (1) the insertion sequence mapped to a single contig with 80–120% of the expected length covered; (2) both flanking sequences mapped to the same contig on the correct strand; and (3) the distance between the mapped flank ends and the insertion coordinates on the contig was within acceptable tolerance. Successfully validated TE insertions were formatted as VCF entries. The final TE-containing assembly was generated using bcftools [65] consensus, which applied the insertions to the reference assembly.

Polishing of draft assemblies was performed in three successive stages using both Oxford Nanopore Technologies (ONT) long reads and Illumina short reads. First, Clair3 polishing with ONT reads [75]. ONT reads were aligned to the draft assembly with minimap2 v2.24 using the map-ont preset, and alignments were sorted and indexed with samtools v1.14. Variants were called with Clair3 v0.1-r12 using the ONT model and the --enable_long_indel option. Homozygous alternate variants (1/1) flagged as PASS with insertion or deletion lengths <350 bp were retained. Filtered variants were applied with bcftools consensus v1.14 [65] to produce the Clair3-polished assembly. The Clair3-polished assembly was then further polished using the PEPPER-Margin-DeepVariant pipeline [76]. ONT reads were re-aligned to the Clair3-polished assembly with minimap2, and variants were called using the ONT R9.4.1 Guppy5 SUP model. Homozygous alternate variants were filtered and applied with bcftools consensus, generating the PEPPER-DeepVariant–polished assembly. For the third round of polishing, Illumina paired-end reads were aligned to the Pepper-Margin-DeepVariant polished assembly with bwa-mem, and duplicates were marked with samtools. Variants were called with DeepVariant v1.1.0 [77] in whole-genome mode, filtered for homozygous alternate alleles, and applied with bcftools consensus to generate the final Illumina-DeepVariant–polished assembly.

### Genome annotation/UCSC hub generation

To annotate the assembled genome and generate a browser hub for visualization in the UCSC Genome Browser [38], we implemented a custom multi-step pipeline integrating gene annotation, repeat masking, read mapping, uniqueness profiling, and variant detection.

#### Assembly preprocessing and hub initialization

Genome assemblies were preprocessed to ensure compatibility with downstream tools. Sequence identifiers containing special characters were sanitized using seqkit v2.3.0, and chromosome size files were generated. Assemblies were converted to 2bit format using faToTwoBit (UCSC Kent utilities [38]) to support hub configuration.

#### Gene annotation strategy

A multi-tiered annotation strategy combined homology-based transfer, transcript alignment, and de novo gene prediction:

- Lift-over annotation: Reference annotations from *Drosophila melanogaster* (dm6) [31, 78] were transferred with Liftoff v1.6.3 [79], which uses sequence alignment to identify homologous loci and project gene models, while resolving structural variations. Pseudogenes were reclassified as mRNA features for consistency in downstream processing.
- Transcript mapping: FlyBase [78] transcript sequences (mRNA, CDS, UTRs, tRNAs, miRNAs, ncRNAs, pseudogenes) were re-aligned to the assembly. Y-linked transcripts were excluded unless explicitly requested.
  o Full-length transcripts and CDS were aligned using BLAT (parameters: -stepSize=5 -fine -q=dna) [80], partitioned for parallel execution. Alignments covering ≥90% of transcript length were retained.
  o Short elements (<50 bp), such as UTRs, were aligned using STAR [81] with a genome index built from the target assembly.
  o BLAT and STAR results were merged, correcting spurious micro-introns (<20 bp) and enforcing accurate CDS boundaries.
  o The final transcript set was exported in bigGenePred format, validated with *genePredCheck*, and indexed (ixIxx) for fast UCSC search integration.
- De novo gene prediction: Long-read ONT RNA-seq and Illumina short-read RNA-seq data were used for ab initio discovery.
  o ONT RNA reads were aligned with minimap2 (splice-aware mode), and Illumina reads with HISAT2 v2.2.1 [82].
  o Transcripts were assembled with StringTie v2.2.1 [83] using mixed-mode analysis (minimum anchor length = 4 bp, minimum junction coverage = 2 reads, minimum transcript coverage = 3.9 reads per base).
- Annotation integration: Gene models from lift-over, transcript mapping, and de novo prediction were merged with a custom algorithm that prioritized evidence-supported annotations and resolved overlaps.

#### Repeat element annotation

Repetitive elements were annotated using RepeatMasker v4.1.2 with the Dfam database [36]. Outputs were converted into bigBed tracks for efficient UCSC visualization.

#### Read mapping and coverage tracks (ONT)

Raw ONT DNA sequences were aligned using minimap2 (-ax map-ont), while ONT direct RNA and cDNA reads were mapped with splice-aware parameters (-ax splice -uf -k14). Alignments were sorted with samtools, and coverage tracks were generated using mosdepth. Normalized depth profiles were converted into bigWig format for direct genome browser display.

#### Genome uniqueness mapping

Uniqueness profiles were generated by sliding-window k-mer analysis. K-mers (25–50 nt) from both strands were generated using seqkit sliding. Single-copy k-mers were remapped to the assembly with Bowtie v1.3.1 [84] under multiple mismatch thresholds (0–3). The 5′ positions of uniquely mapped k-mers were extracted, converted to bigBed format, and organized into UCSC tracks representing regions of unique mappability.

#### Variant detection

- ONT-based variants: ONT DNA reads were aligned with minimap2 (-Lax map-ont --secondary=no). SNPs and indels were identified with Clair3 [75] and PEPPER-DeepVariant [76], while structural variants were detected with Sniffles v1.012a [72] and cuteSV [85]. Variants were processed with *bgzip* and *tabix*, stratified by zygosity, and summarized with rtg vcfstats (https://github.com/RealTimeGenomics/rtg-tools). Outputs were integrated as vcfTabix tracks.
- Illumina-based variants: Illumina reads were mapped with BWA-MEM [67] followed by filtering (samtools view -F 256 [65]), mate-pair correction (*samtools fixmate*), coordinate sorting (*samtools sort*), and duplicate marking (*samtools markdup*). Variants were called using DeepVariant [77] (WGS model), processed identically to ONT calls, and visualized in the UCSC hub.

#### UCSC hub construction

All annotation and analysis outputs (gene models, repeats, coverage, uniqueness, and variants) were converted into UCSC-compatible formats (bigWig, bigBed, bigGenePred, vcfTabix). Hub configuration files (*hub.txt, genomes.txt, trackDb.txt*) were automatically generated with metadata, methodology notes, and visualization defaults. Tracks were grouped and hierarchically organized to facilitate comparative exploration of genome structure, expression, and variation.

### 3′ UTR isoform refinement using ONT direct RNAseq reads

We developed a computational pipeline to refine 3′ UTR annotations and quantify 3′ UTR isoform usage by leveraging 3′ termini from Oxford Nanopore Technologies (ONT) direct RNA-seq reads. Our approach involved five key steps: (i) anchoring 3′ UTRs to coding sequence (CDS) stop codons, (ii) defining a conservative search window per transcript to exclude downstream genic and repetitive interference, (iii) identifying candidate 3′ ends from ONT read termini, (iv) tiling and quantifying local 3′ end signal, and (v) applying sequential filters to remove artifacts and low-confidence sites, ultimately yielding high-confidence 3′ UTR isoforms. Transcriptional stop codon positions, derived from expressed gene models, served as the initial anchors for 3′ UTR definition. Subsequently, UTR search intervals were established downstream of stop codons for sense strands and upstream for antisense strands. These windows were refined by considering the longest observed UTRs per gene and by intersecting with expressed annotations and repeat elements to prevent interference from unrelated genomic features. To identify candidate 3′ ends, ONT direct RNA-seq read 3′ termini were extracted from alignments. These positions were then grouped into "tiles" based on local read support, and each tile was quantified for total reads, peak reads, tile-specific reads, and fractional contribution.

For primary filtering, two strategies were employed:

- Continuous “RAMP-down” selection: This method iteratively retained tiles with significant read support, prioritizing pronounced drops in abundance consistent with major 3′ ends.
- Fraction-based filtering: Tiles were retained if they met predefined minimum read count thresholds and either a minimum fractional contribution or a minimum absolute read count.

Finally, to reduce redundancy, closely spaced minor peaks were either merged or discarded if they were within a defined proximity of a more abundant neighbor.

### Genome statistic comparison

Assembly quality and contiguity (Nx statistics) were assessed with QUAST [86]. Both the *Drosophila melanogaster* reference genome (dm6) and the OSC_r1.01 assembly were analyzed using the FlyBase reference annotation as a feature set. QUAST was run in large-genome mode with scaffold splitting, conserved gene identification, and eukaryote-specific settings (--large --features flybase.gff --split-scaffolds --conserved-genes-finding --eukaryote).

### BUSCO

Assembly completeness was evaluated using BUSCO v5 [87] with the *drosophila_odb12* lineage dataset in genome mode. BUSCO summary statistics and completeness plots were generated from the resulting output files.

### Whole-genome alignment and synteny visualization

Pairwise whole-genome alignments between each target assembly and the *Drosophila melanogaster* reference genome (dm6) were performed using NUCmer [88] with the parameters --maxmatch -c 100 -b 500 -l 50, which allow for sensitive detection of maximal exact matches and robust alignment of complex regions. Filtered alignments were converted to PAF format using *paftools delta2paf * [89] to enable downstream analysis and visualization. Repetitive versus unique alignments were classified by calculating mapping multiplicity of both query and reference intervals: alignments with multiple mappings were annotated as “multi”, whereas those mapping uniquely were retained as “uniq”. Dot plots were generated using a modified version of *paf2dotplot* (https://github.com/moold/paf2dotplot) that incorporated repeat annotations for color-coding and a custom color-scheme. Genome-wide synteny plots were created under standard filtering thresholds (minimum alignment length of 5 kb; minimum reference span of 100 kb). For targeted locus analysis, higher-resolution dot plots (minimum alignment length 1 kb; minimum reference span 1 kb) were produced, focusing on the extended *flamenco* region on chromosome X. For detailed analysis of the *flamenco* piRNA cluster, locus-specific sequences were extracted from dm6 and OSC_r1.01. Pairwise NUCmer alignments were performed, filtered with delta-filter, and analyzed for structural rearrangements using SyRI (--nosnp --tdgaplen 10000) [90]. Local synteny plots were generated with plotsr (-s 5) [91] to visualize rearrangements within the *flamenco* cluster. For detailed analysis of the *flamenco* piRNA cluster, locus-specific sequences were extracted from dm6 and OSC_r1.01. Pairwise NUCmer alignments were performed, filtered with delta-filter, and analyzed for structural rearrangements using SyRI (--nosnp --tdgaplen 10000) [90]. Local synteny plots were generated with plotsr (-s 5) [91] to visualize rearrangements within the *flamenco* cluster.

### Hi-C data analysis

Hi-C sequencing reads were mapped to the OSC_r1.01 genome assembly using BWA-MEM [67] with the -P option for Hi-C paired-end data. Alignments were processed using samtools [65] and parsed with pairtools (v.1.1.3) [92]. Duplicate fragments were filtered with pairtools dedup to generate sets of valid pairs, duplicates, unmapped reads, and summary statistics. Hi-C sequencing reads were mapped to the OSC_r1.01 genome assembly using BWA-MEM [67] with the -P option for Hi-C paired-end data. Alignments were processed using samtools [65] and parsed with pairtools (v.1.1.3) [92]. Duplicate fragments were filtered with pairtools dedup to generate sets of valid pairs, duplicates, unmapped reads, and summary statistics. For chromosome-scale contact map construction, valid Hi-C pairs were converted into a balanced matrix format using Cooler (v.0.10.4) [93]. Bin intervals were generated at 1 kb resolution with cooler makebins. Matrices were built with cooler cload pairs and balanced using cooler balance with a maximum median absolute deviation (MAD) threshold of 5. To enable multi-resolution analysis of genome architecture, balanced matrices were zoomified with cooler zoomify at 1 kb, 2 kb, 5 kb, 10 kb, 25 kb, 50 kb, and 100 kb resolutions. The resulting.mcool files were used for downstream visualization and quantitative analysis of Hi-C contact patterns. Hi-C contact matrix was visualized using HiGlass [94]. For chromosome-scale contact map construction, valid Hi-C pairs were converted into a balanced matrix format using Cooler (v.0.10.4) [93]. Bin intervals were generated at 1 kb resolution with cooler makebins. Matrices were built with cooler cload pairs and balanced using cooler balance with a maximum median absolute deviation (MAD) threshold of 5. To enable multi-resolution analysis of genome architecture, balanced matrices were zoomified with cooler zoomify at 1 kb, 2 kb, 5 kb, 10 kb, 25 kb, 50 kb, and 100 kb resolutions. The resulting.mcool files were used for downstream visualization and quantitative analysis of Hi-C contact patterns. Hi-C contact matrix was visualized using HiGlass [94]. 

### SNP (Single Nucleotide Polymorphism) detection

Illumina DNA-seq reads were aligned to the genomes using BWA-MEM [67], followed by sorting, indexing, and filtering for uniquely mapped reads with samtools (multi mapping alignments were excluded by using option -q 1) [65]. Variant calling was performed using DeepVariant (WGS model) [77] with assembly-specific references to generate VCF outputs and summarized with rtg vcfstats. In brief Deepvariant encodes read pileups around each candidate variant as image-like tensors and classifies genotypes (0/0, 0/1, 1/1) using a trained convolutional neural network (CNN). The CNN outputs genotype probabilities based on learned patterns of allele balance, base quality, mapping quality, and strand bias, and assigns the most likely genotype accordingly. To quantify local SNP density, assemblies were partitioned into 1-kb windows using bedtools and homozygous and heterozygous SNP sets were separated using bcftools. Genome-uniqueness-filtered (>50% genome unique) windows were intersected with homozygous and heterozygous SNPs to obtain per-window counts, enabling assessment of genome-wide and locus-specific variant distributions.

Loss-of-heterozygosity (LOH) regions were identified from the per-window heterozygous SNP counts described above. Consecutive 1-kb windows with fewer than 5 heterozygous SNPs were merged into contiguous blocks using a custom awk script. Only blocks exceeding 500 kb in length were retained as high-confidence LOH regions

### SV (Structural Variant) detection

Oxford Nanopore DNA reads were aligned to assemblies using minimap2 (-Lax map-ont --secondary=no) and sorted/indexed with samtools. Structural variants (SVs) were identified using Sniffles v2 [72, 95], which computes genotype likelihoods based on a binomial distribution of observed variant and reference reads, with default settings and summarized with rtg vcfstats. To classify SVs as transposable element (TE)-associated or not, insertion and deletion sequences >50 bp were extracted from VCF entries and aligned against a curated TE consensus library (based on RepBase [73]) using minimap2. SVs with ≥80% sequence coverage from TE alignments were annotated as TE-related. 

### Inter-genome SV detection

Structural variants (SVs) were identified by comparing the OSC genome assembly to the reference dm6 genome using minimap2 (v2.24) with parameters optimized for assembly-to-assembly alignment (-a -x asm5 --cs -r2k). Alignments were sorted and indexed using samtools (v1.15). SVs were called using SVIM-asm [96] in haploid mode.

To determine the zygosity of identified SVs in the OSC cell line, Oxford Nanopore Technologies (ONT) long-read sequencing data was mapped to the OSC assembly using minimap2 with ONT-specific parameters (-Lax map-ont --secondary=no). Genotyping was performed using Sniffles v2, which computes genotype likelihoods based on a binomial distribution of observed variant and reference reads, with the --genotype-vcf option to assess whether SVs were homozygous or heterozygous in the original sequencing data.

SVs larger than 50 bp were extracted and classified for transposable element (TE) content. SV sequences were aligned to a comprehensive TE consensus library (based on RepBase [73]) using minimap2 (--paf-no-hit). SVs were classified as TE-containing if ≥80% of the SV sequence aligned to TE consensus sequences (based on RepBase [73]). TE classifications were merged using bedtools to handle overlapping alignments, and SVs were categorized as containing single TEs, multiple TEs, or no TE content.

### Chromatin state analysis at TE insertions

ChIPseq libraries were pre-process using the labs NGS data pre-processing pipeline (https://github.com/BrenneckeLab/AnnotationPipeline). In brief

Full-length TE insertions were identified by aligning TE consensus sequences (based on Repbase [73]) to the genome assembly using BLAT [80]. High-confidence insertions were defined as those with near end-to-end alignment (query start <50 bp, query end >query size-50 bp) and ≥80% sequence identity. H3K9me3 ChIP-seq signal was analyzed around TE insertion sites using deepTools (v3.5.4) [97]. Signal matrices were computed using computeMatrix scale-regions with 5 kb flanking regions and 10 bp bins. Only TE insertions in euchromatic regions were analyzed, defined by chromosome coordinates excluding heterochromatic regions. Heatmaps were generated using plotHeatmap with k-means clustering (k=3) to identify distinct chromatin patterns. TEs were classified into “Piwi regulated” and “Piwi independent “ by a cutoff of >2.5x log2 fold-deregulation as determined in the RNAseq DGE analysis. 

### Clonal validation of TE insertions

To validate TE insertions in an independent clonal OSC line, we developed a flank-based mapping strategy. For each insertion, 200 bp flanking sequences were extracted from the upstream and downstream TE-genome borders using bedtools getfasta. These flanks were combined with the TE sequence to create a specialized mapping reference.

Clonal DNA-seq reads (ChIP input) were first filtered for genome-uniqueness by mapping to the full OSC assembly with Bowtie (-m 1 -v 2). These unique reads were then aligned to the flank-combined reference. To ensure reads were anchored in genomic sequence rather than mapping solely within the TE body, we required reads to extend at least 20 bp into the flanking genomic region. Per-position coverage was calculated using bedtools genomecov and stratified by TE family, zygosity, and H3K9me3 enrichment group.

To determine the theoretical limit of detection, we performed a parallel "genome uniqueness" analysis. We generated all possible 50-mer sequences from the OSC assembly, retained those that occurred exactly once, and processed them through the same flank-mapping pipeline. This provided a baseline for mappability at each TE-genome border, identifying regions where local sequence complexity allows for unambiguous validation.

### RNAseq analysis

Illumina reads were processed by removing 3′ adaptors with cutadapt [98], trimming the first five nucleotides, and filtering for length (>18 nt) and sequence complexity (bbduk, entropy = 0.35, entropy window = 18, k = 4) [99]. Reads were first aligned to *Drosophila* rRNA precursor sequences and the mitochondrial genome using bowtie [84]. Remaining unmapped reads were mapped to the *OSC_r1.01* genome using STAR in two-pass mode with splice-junction support (--alignEndsType Local, --outFilterType BySJout, --alignSJoverhangMin 15, --alignSJDBoverhangMin 1, --outFilterMismatchNmax 1, --outFilterMultimapNmax 1000, --winAnchorMultimapNmax 2000) [81]. Alignments were separated into uniquely and all-mapping fractions. For visualization, HOMER [100] was used to generate tag directories (makeTagDirectory … -format bed -keepAll -single -fragLength given) and UCSC-compatible tracks (makeUCSCfile … -fsize 1e20 -strand +/- -fragLength given -noadj -normLength 0) were generated per strand. Counts were normalized to 10M uniquely aligned reads and final BigWig tracks were created with bedGraphToBigWig (Kent Utilities) [38], producing strand-specific (sense and antisense) coverage profiles for genome browser display. For differential gene expression (DGE) analysis gene and transposon expression was first quantified by using Salmon [101] on a target file containing *Drosophila* reference transcripts and TE sequences in sense an antisense orientation (--dumpEqWeights --seqBias --gcBias --useVBOpt --numBootstraps 100 -l SF --incompatPrior 0.0 --validateMappings). Salmon results were imported into R using tximport [102]. Gene-level counts were assembled into a DESeq2 [103] dataset with sample metadata, and genes with fewer than 10 total counts across all samples were filtered out. Conditions were modeled using ~ condition, and reference genotypes were defined by re-leveling as appropriate. Variance stabilization was applied using either variance stabilizing transformation (VST) or regularized log transformation (vst, blind = FALSE), depending on the number of detected genes. Differential expression analysis was conducted using DESeq2 with independent filtering, Benjamini–Hochberg correction (α = 0.05), and log2 fold change shrinkage with apeglm [104]. Additional gene-level GeTMM value statistics have been calculated using edgeR [105].

### ChIPseq analysis

ChIP-seq libraries were pre-processed using the lab’s NGS data pre-processing pipeline (https://github.com/BrenneckeLab/AnnotationPipeline). In brief, Illumina reads were processed by removing 3′ adaptors with cutadapt [98] and filtering for minimal length (>18 nt) and sequence complexity (bbduk, entropy = 0.35, entropy window = 18, k = 4) [99]. Reads were first aligned to Drosophila rRNA precursor sequences and the mitochondrial genome using Bowtie (-v 1 -a) to remove contaminating reads. The remaining reads were then aligned to the genome to identify unique mappers using Bowtie (-v 1 -m 1 --best --strata). Reads that did not align uniquely were subsequently processed separately as multi-mappers using Bowtie (-v 1 -a --best --strata). Tag directories were created using HOMER [100](makeTagDirectory), and unstranded BigWig tracks were generated using HOMER’s makeUCSCfile followed by conversion with bedGraphToBigWig.Tracks were normalized to 10 million reads in the input fasta file. Results were organized into UCSC track hubs for genome browser visualization.

### sRNA-seq analysis

Small RNA-seq libraries were pre-processed using the laboratory’s standardized NGS data pipeline (https://github.com/BrenneckeLab/AnnotationPipeline). Illumina reads were first processed to remove 3′ adaptors using cutadapt [98]. For libraries using sRBC barcoded adaptors, an additional two nucleotides following the barcode were trimmed. To account for library construction artifacts, four random nucleotides were trimmed from each end of the reads. Finally, reads were filtered for a length of (>18 nt; <=35nt) and for sequence complexity using bbduk (entropy = 0.35, entropy window = 18, k = 4) [99]. Reads were first aligned to *Drosophila* rRNA precursor sequences and the mitochondrial genome using bowtie (-v 1 -a) [84]. The remaining reads were then aligned to the genome to identify unique mappers using Bowtie (-v 1 -m 1 --best --strata). Reads that did not align uniquely were subsequently processed separately as multi-mappers using Bowtie (-v 1 -a --best --strata). For visualization, HOMER [100] was used to generate tag directories (makeTagDirectory … -format bed -keepAll -single -fragLength given) and UCSC-compatible tracks (makeUCSCfile … -fsize 1e20 -strand +/- -fragLength given -noadj -normLength 0) were generated per strand. Counts were normalized to 1M miRNAs and final BigWig tracks were created with bedGraphToBigWig (Kent Utilities) [38], producing strand-specific (sense and antisense) coverage profiles for genome browser display. For transposon histogram analysis, piRNA reads longer than 23 (>23nt; <=35nt) nucleotides were aligned to transposable element consensus sequences (based on RepBase [73]) using bowtie (*--best --strata -f -v 2 -a*), permitting multiple mappings. Alignments were subsequently filtered to retain reads that mapped either uniquely or at most twice to a single transposon consensus, to account for long terminal repeat (LTR) redundancy. Reads mapping twice were assigned a fractional count of 0.5 to each location.

### PROseq analysis

Paired-end Illumina reads were processed using the laboratory’s standardized NGS data pipeline (https://github.com/BrenneckeLab/AnnotationPipeline). The first mate was removed, and 5′ adaptors were clipped from the second mate using cutadapt [98]. Reads were then filtered for a minimum length of 18 nt and for sequence complexity using bbduk (entropy = 0.35, entropy window = 18, k = 4) [99]. To remove contaminating sequences, reads were first aligned to *Drosophila* rRNA precursor sequences and the mitochondrial genome using Bowtie (-v 1 -a). Remaining unmapped reads were mapped to the OSC_r1.01 genome using STAR [81] in two-pass mode with splice-junction support (--alignEndsType Local, --outFilterType BySJout, --alignSJoverhangMin 15, --alignSJDBoverhangMin 1, --outFilterMismatchNmax 1, --outFilterMultimapNmax 1000, --winAnchorMultimapNmax 2000). Alignments were separated into uniquely and all-mapping fractions. For visualization, HOMER [100] was used to generate tag directories (makeTagDirectory … -format bed -keepAll -single -fragLength given) and UCSC-compatible tracks (makeUCSCfile … -fsize 1e20 -strand +/- -fragLength given -noadj -normLength 0) were generated per strand. Counts were normalized to 10M uniquely aligned reads and final BigWig tracks were created with bedGraphToBigWig (Kent Utilities) [38], producing strand-specific (sense and antisense) coverage profiles for genome browser display.

### *flamenco* splicing analysis

The OSC_r1.01 genome was indexed with STAR [81], and paired-end RNA-seq data were split into single reads, reoriented, mapped as single-end data with STAR using --alignEndsType EndToEnd and two-pass mapping. Uniquely aligned reads were extracted with samtools (NH:i:1) [65] and indexed. Spliced alignments were identified from CIGAR strings containing “N” operators and converted to BED12 [66]. Splice junctions were collapsed with bedtools groupby, and junction counts were combined with flanking coverage to produce UCSC “interact” format tracks. Additional scoring was introduced by quantifying mean read coverage in a 1 nt upstream window of donor sites relative to junction read support. Junctions with at least three supporting reads and upstream coverage greater than ten were retained and scaled to UCSC scores (0–1,000). Final splice junction interact tracks were converted to BigBed with bedToBigBed using a custom interact.as schema, allowing direct genome browser visualization of putative splicing within *flamenco*.

### *flamenco* silencing by tethering - analysis

The initial 800 kb of the *flamenco* locus and additional control regions were subdivided into 100 bp non-overlapping tiles, and uniqueness was estimated from 25-mer mappability tracks (1 mismatch allowed). For each tile, uniquely mappable positions and percent unique coverage were calculated. In parallel, the entire X chromosome was partitioned into 1-kb tiles on both strands. Tiles were intersected with 25-mer uniqueness tracks, and only those with >50% uniquely mappable positions were retained. Small RNA alignments from individual libraries were then intersected with the tiled features, retaining only uniquely mapped reads. Counts were normalized per library to 1 million miRNAs. For each tile, normalized counts and counts per unique position were calculated, and final count tables were compiled across libraries to allow quantitative comparisons of occupancy along *flamenco*, additional control regions, and across the full X chromosome. Small RNA count tables derived from tiled genomic regions were imported into R. Because of their close similarity, the 6- and 7-day post-transfection samples were treated as replicates. Tiles with ≤50% uniquely mappable positions and libraries outside the relevant time points were excluded. For fold-change analysis, tiles with insufficient coverage (≤1 count for locus-specific analysis or ≤75 counts for chromosome-wide analysis) were filtered out. Fold changes were then calculated by dividing normalized values of treatment samples (IDR-tethered) by their corresponding controls (GAL4).

### TE cluster coverage analysis

Transposable element (based on RepBase [73]) sequences were indexed using bowtie. small RNA reads longer than 23 nucleotides were truncated to 25 nucleotides and aligned to TE consensus sequences using bowtie (-a -M 1 --best –strata) [84]. Alignments were converted to BED format and strand-specific coverage was calculated using bedtools genomecov (-strand +/-) [65, 66]. For cluster coverage analysis, 25-mer sequences were extracted from piRNA cluster coordinates using bedtools getfasta and seqkit sliding (-s 1 -W 25) for both OSC assembly and reference genome (dm6). These 25-mers were aligned to TE consensus sequences using bowtie (-v 1 -a), allowing all valid alignments, and converted to strand-specific bedGraph format. Coverage tracks from small RNAs and cluster-derived sequences were merged using join to produce position-wise comparison tables. Coverage data were imported into R and converted to binary format using dynamic thresholds (cluster coverage >0, small RNA coverage >10% of mean TE coverage). Overlap scores were then calculated independently for sense and antisense strands. Specifically, for each TE and strand:

$$\text{Overlap score}=\frac{\text{Number of positions with}\; (\mathrm{sRNA}=1\;\wedge \mathrm{cluster=1})}{\text{Total number of positions with}\; (\mathrm{sRNA}=1)}$$

This value quantifies the fraction of small RNA–covered positions along a TE that are also represented by cluster-derived sequences. TE consensus elements with peak small RNA coverage <1000 reads or mean coverage <220 reads were excluded.

### Statistical analysis and visualization

All statistical analyses and visualizations were performed in R (v4.3.2) [106]. Data wrangling and plotting were primarily carried out with the tidyverse collection (including *dplyr*, *ggplot2*) [107–109], with figure assembly using cowplot [110] and patchwork [111]. Interactive data exploration was supported by *plotly* [112] and formatted with the *scales* [113] package. Additional visualization packages included *ggbeeswarm* [114] *ggforce* [115], *ggh4x* [116], *khroma* [117], ggrastr [118] and *paletteer* [119] for custom themes, categorical palettes, and swarm/strip plots. JSON-based data handling employed *jsonlite* [120].

## Supplementary Information

Additional file 1. This document contains Supplemental Figures S1–S4, Supplemental Tables S1, S3, and S4 and supplemental references.

Additional file 2. Comprehensive inventory of sequencing datasets. This table provides metadata and accessions for all libraries generated in this study, as well as external datasets integrated into the analysis.

## Data Availability

Genome assembly [121], Illumina and Nanopore genomic DNA sequencing [122–124] Hi-C [125], RNA-seq [126], small RNA [127], and PRO-seq [128] data generated in this study have been deposited under BioProject PRJNA1338230. VCF files listing SV and SNP data shown in Figs. 1 and 3 have been deposited on Zenodo under https://doi.org/10.5281/zenodo.20059415 [129]. Published datasets used in this manuscript include Nanopore direct RNA-seq: PRJNA1236369 [130, 131], STARR-seq and DHS-seq: PRJNA175267 [37, 132], Illumina RNA-seq: PRJNA724718 [29, 133] and ChIP-seq: PRJNA724736 [34, 134], PRJNA1381473 [34, 135], PRJNA178497 [24, 136], PRJNA724736 [29, 137]. A detailed inventory of all datasets, including published data, is provided in Additional file 2: Table S2. The assembly is available via a UCSC Genome Browser hub with annotations and data tracks at https://brenneckelab.imba.oeaw.ac.at/Publication_Data/2026_Handler_OSC-genome/ and as a hosted UCSC session for direct browsing: https://genome-euro.ucsc.edu/s/Brennecke%2DLab/OSC_r1.01_Handler_et.al._2025. The source code for genome assembly and subsequent analyses is hosted on GitHub within a master repository linking to all modular components (https://github.com/BrenneckeLab/Handler_2026-OSC-genome). All code modules are released under the MIT license. Version-controlled snapshots of the specific code versions used in this study have been deposited in Zenodo [106, 138–144].
