# Supplementary material for: A reference genome for cultured Drosophila ovarian somatic cells enables studies of transposon and piRNA biology
Source: Genome Biol. 2026 May 22;27:227. doi: 10.1186/s13059-026-04113-y (PMC13371464; doi:10.1186/s13059-026-04113-y)
Supplement: Supplementary file 1 — Additional file 1. This document contains Supplemental Figures S1–S4, Supplemental Tables S1, S3, and S4 and supplemental references. [file 13059_2026_4113_MOESM1_ESM.pdf]

Supplementary Materials for

The Drosophila OSC Genome: A Resource for Studies of Transposon and piRNA Biology

Dominik Handler & Julius Brennecke

Institute of Molecular Biotechnology of the Austrian Academy of Sciences (IMBA), Vienna BioCenter (VBC); Vienna, 1030, Austria.

correspondence: dominik.handler@imba.oeaw.ac.at; julius.brennecke@imba.oeaw.ac.at

The Supplemental materials include:

Figures S1 to S4

Supplementary Table 1-4

Supplementary References

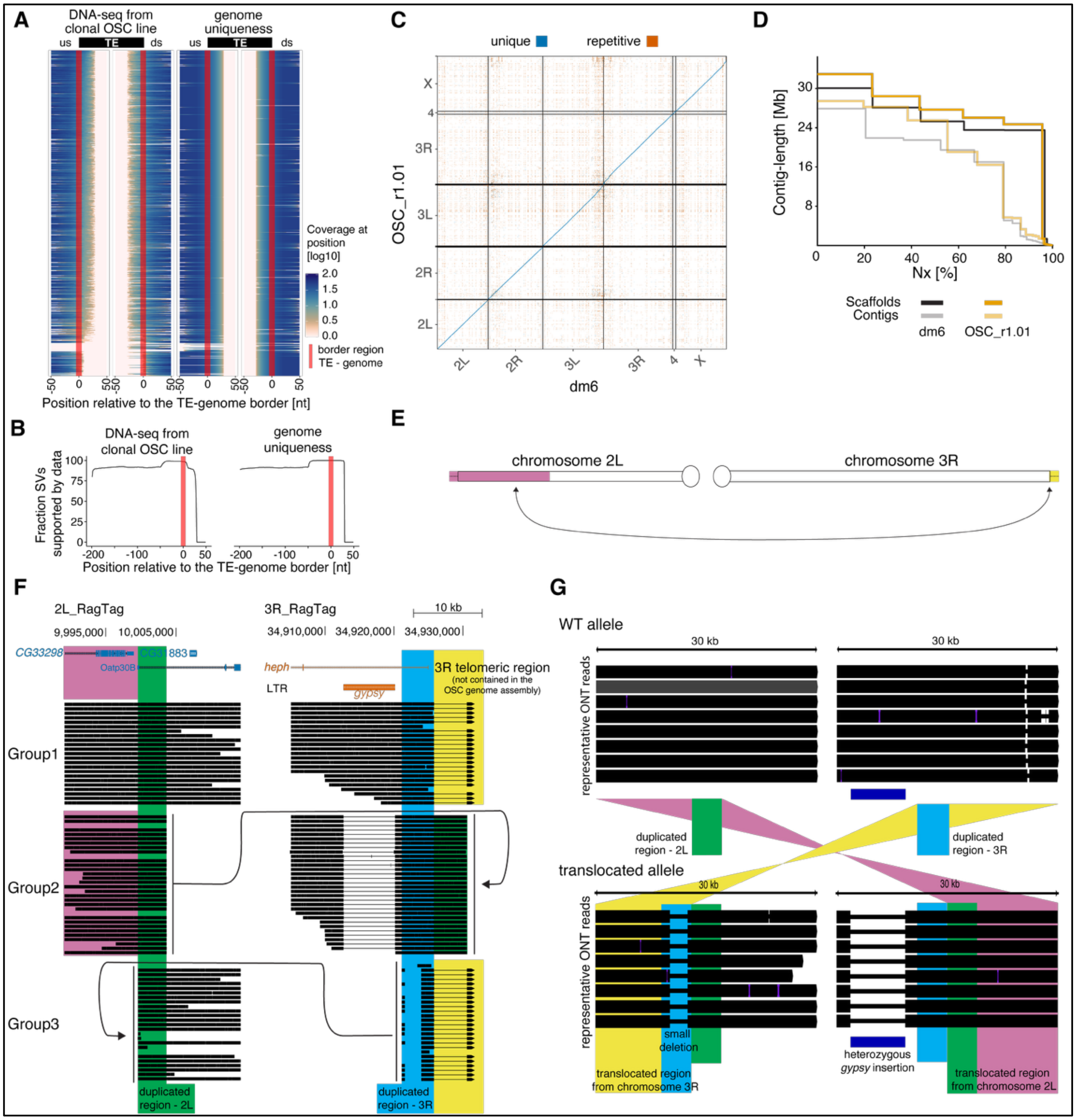

Figure S1

(A) Validation of OSC_r1.01 transposon insertions in a clonal OSC line. Heatmap showing DNA-seq read coverage from a clonal OSC derivative at the genomic borders of transposable element (TE) insertions present in the OSC_r1.01 assembly. For each insertion, coverage is shown for the upstream (us) and downstream (ds) flanking regions relative to the TE-genome border. Only genome-uniquely mapping reads are shown; the corresponding genome uniqueness heatmap (right panel) indicates the regions that are accessible for unambiguous mapping. Continuous read coverage spanning the boundary regions confirms the presence and fixation of these insertions in the clonal line. **(B)** Quantification of the TE

insertion presence based on the data shown in (A). An insertion is counted as present in the clonal line if genome-unique read coverage is detected at either the upstream or downstream TE-genome flank. The fraction of TE insertions supported by the clonal data is plotted relative to the insertion boundary. The corresponding genome uniqueness plots (right) indicate the theoretical limit of detection based on sequence mappability. **(C)** Dot plot comparing the OSC_r1.01 assembly with dm6, colored by alignment uniqueness. Alignments shorter than 5 kb were excluded. **(D)** Contig NG(x) plots of OSC_r1.01 and dm6 assemblies shown as scaffolds and as contigs after breaking at assembly gaps. **(E)** Schematic representation of the reciprocal translocation in OSCs, involving ~10 Mb from distal chromosome 2L and the distal end of chromosome 3R. **(F)** Nanopore read alignments confirming the heterozygous 2L–3R translocation. Group 1: reads supporting the intact, non-translocated alleles. Group 2: reads spanning the junction between the distal segment of chromosome 2L and the chromosome body of 3R (dm6 coordinates: 2L:9,525,366 and 3R:31,970,603). Group 3: reads spanning the junction between the chromosome body of 2L and the translocated distal segment of 3R (dm6 coordinates: 2L:9521264 and 3R:31,966,484). The presence of a ~4 kb segment of 2L in all three read groups (green) indicates a segmental duplication associated with the rearrangement. (**G**) Representative Oxford Nanopore reads spanning the regular (top) and translocated (bottom) alleles. In the regular configuration, reads map contiguously to chromosomes 2L and 3R. In the translocated allele, long reads span the reciprocal junctions between distal 2L and terminal 3R, confirming the heterozygous rearrangement. Colored blocks denote the duplicated segments and translocated regions as indicated in panel F.

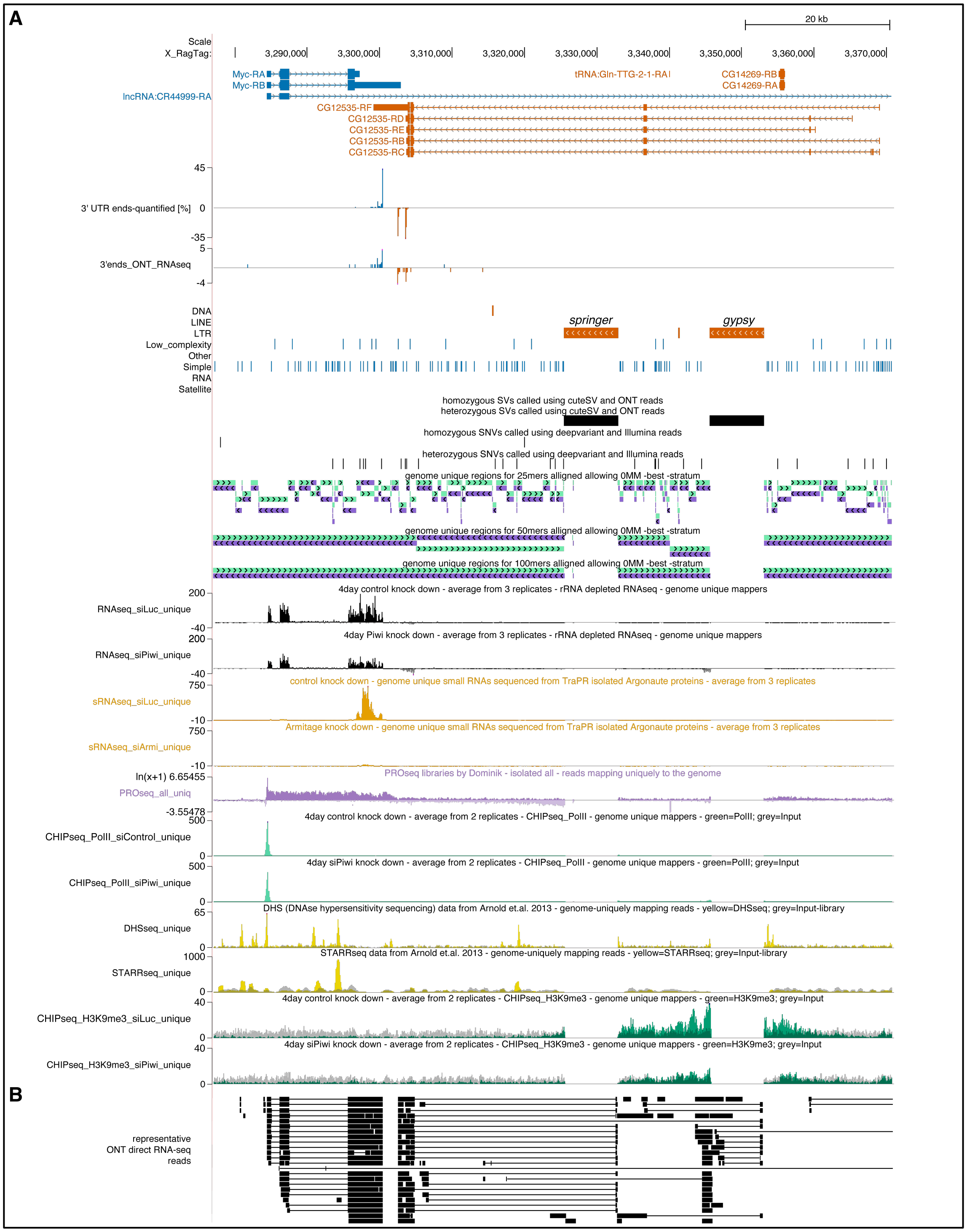

Figure S2

(A) Genome browser view of the *Myc* and *CG12535* locus in OSC_r1.01, as exported from the UCSC Genome Browser. STARR-seq, DHS-seq, ChIP-seq, PRO-seq, and RNA-seq genome-unique signals are shown as coverage per 10 million reads; genome-unique small RNA coverage is normalized to 1M miRNA reads; data are displayed as the average of 3 biological replicates. PRO-seq signal is displayed

as $\ln(x+1)$-transformed coverage. ChIP-seq is shown as an overlay of ChIP (green) and ChIP input (grey). STARR-seq and DHS-seq are shown as an overlay of experiment (yellow) and input (grey). **(B)** Shown are representative Oxford Nanopore (ONT) direct RNA-seq read alignments.

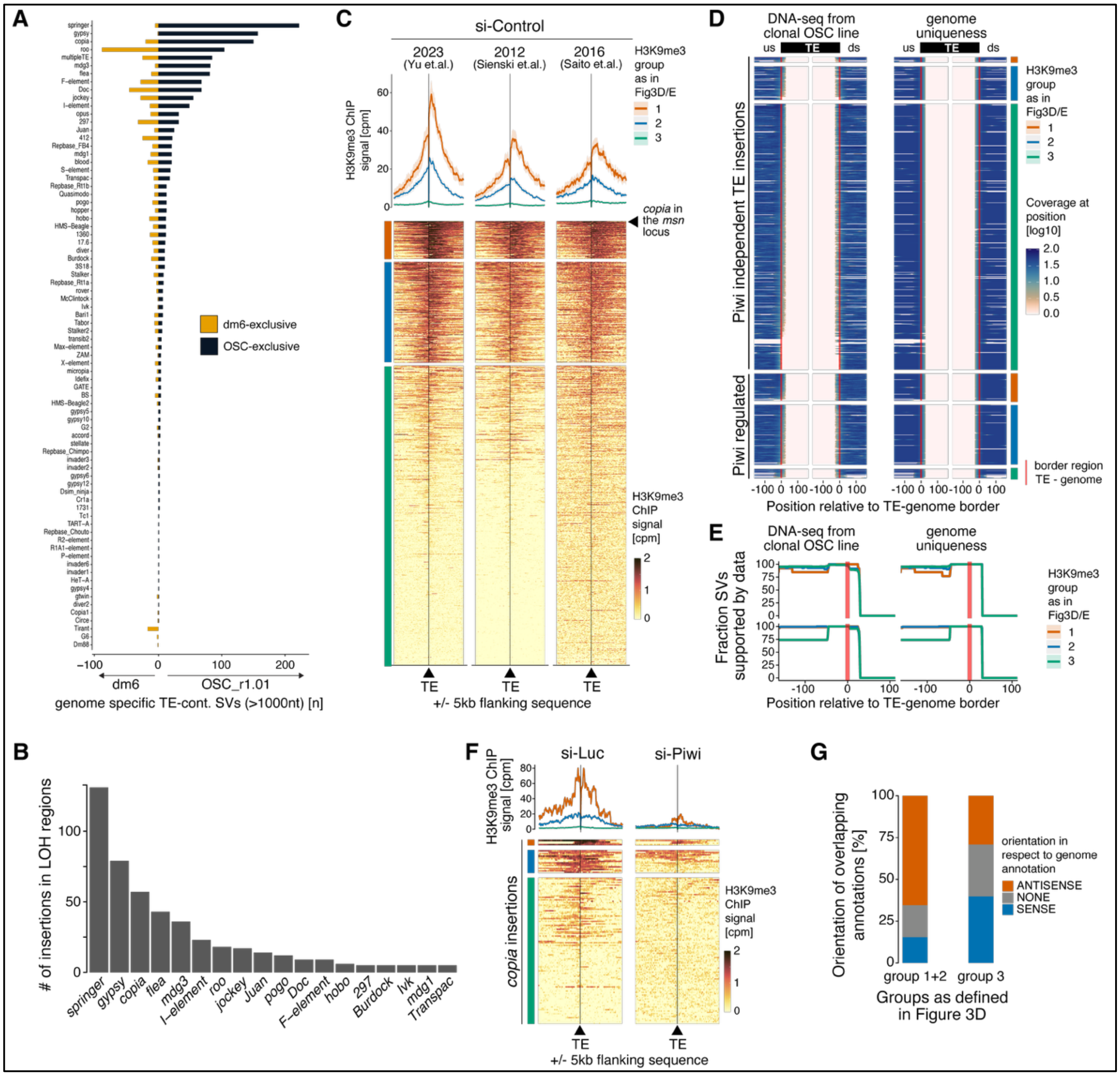

Figure S3

(**A**) Structural variants (SVs) containing >80% transposable element content, shown per transposon family. For each element, dm6-specific insertions are displayed to the left and OSC_r1.01-specific insertions to the right. (**B**) Number of OSC_r1.01-specific heterozygous TE insertions located in LOH regions as defined in Figure 1G. (**C**) Heatmaps of H3K9me3 ChIP-seq signal ±5 kb around full-length (>90% of consensus length) transposon insertions in 3 different unperturbed control experiments. Clustering and sorting identical with Figure 3D. (**D**) Validation of OSC_r1.01 transposon insertions in a clonal OSC line. Heatmap showing DNA-seq read coverage from a clonal OSC derivative at the genomic borders of transposable element (TE) insertions identified in the OSC_r1.01 assembly. For each insertion, coverage is shown for the upstream (us) and downstream (ds) flanking regions relative to the TE-genome

boundary. Only genome-uniquely mapping reads were analyzed; the corresponding genome uniqueness heatmap (right panel) indicates the regions that are accessible for unambiguous mapping. Continuous read coverage spanning the boundary regions confirms the presence and fixation of these insertions in the clonal line. The data are stratified into Piwi-independent and Piwi-regulated TEs and further subdivided by H3K9me3 enrichment groups as defined in Figure 3D. (**E**) Quantification of the TE insertion presence based on data shown in (D). An insertion is counted as present in the clonal line if genome-unique read coverage is detected at either the upstream or downstream TE-genome flank. The fraction of TE insertions supported by the clonal data is plotted relative to the insertion border, stratified by H3K9me3 enrichment groups as defined in Figure 3D. The corresponding genome uniqueness plots (right) indicate the theoretical limit of detection based on sequence mappability. (**F**) Heatmaps of H3K9me3 ChIP-seq signal ±5 kb around full-length (>90% of consensus length) *copia* insertions in control (left) and *piwi*-depleted (right) conditions. Clustering and sorting identical with Figure 3D. (**G**) Quantification of *copia* insertion orientation relative to overlapping expressed transcript orientation, stratified by H3K9me3 groups as in Figure 3D (with groups 1 and 2 merged into a single bar).

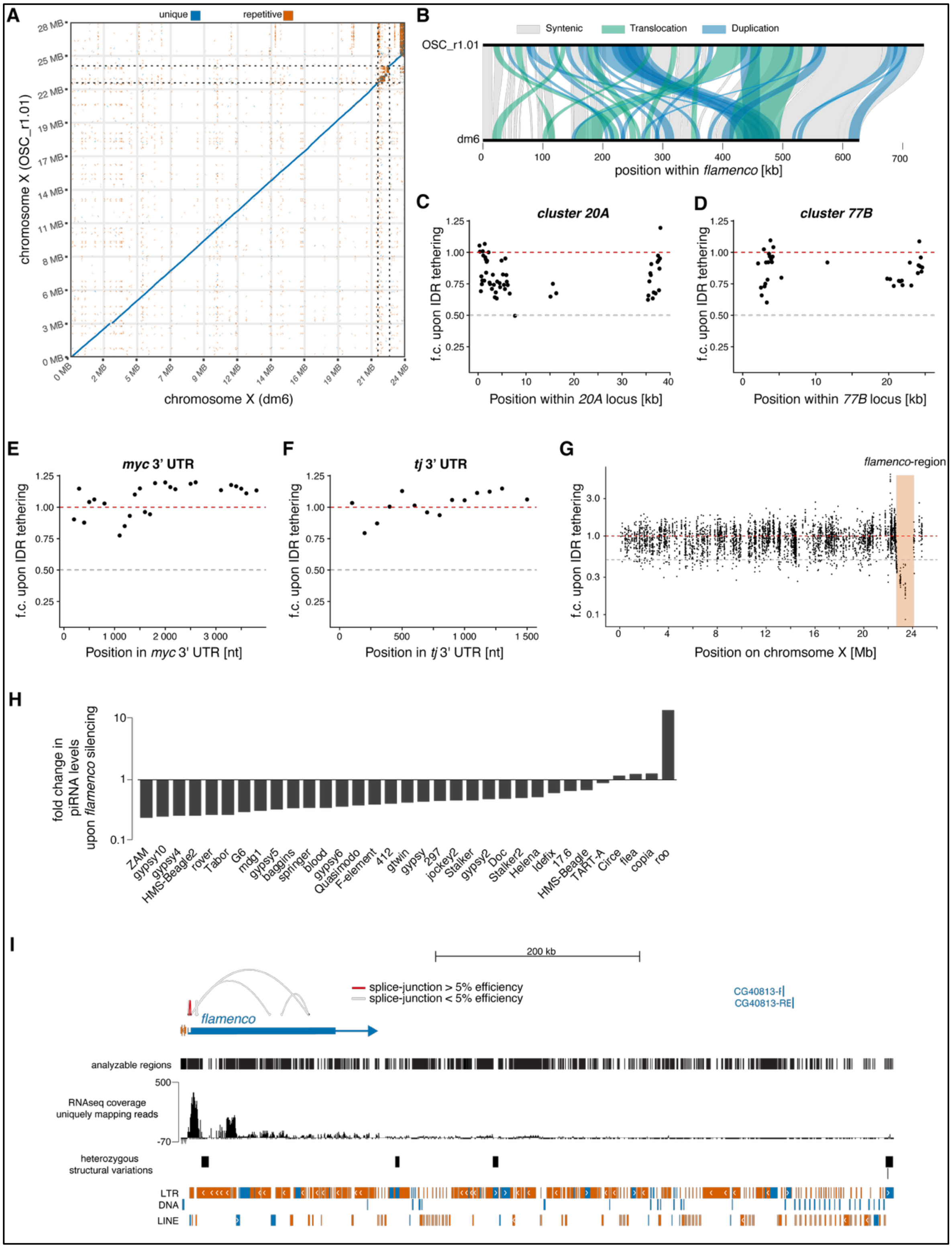

Figure S4.

(**A**) Dot plot comparing the X chromosome of the OSC_r1.01 assembly with dm6, colored by alignment uniqueness. Alignments shorter than 5 kb were excluded. The extended *flamenco* locus spanning the

region from *DIP1* to *CG14621* is indicated by dashed lines in both assemblies. (**B**) Synteny map of the *flamenco* loci in the dm6 and OSC_r1.01 assemblies. Syntenic regions, translocations, and duplications are highlighted in color; regions without synteny are left blank. (**C-F**) 1-kb tile analysis of fold changes in small RNA levels upon repressor tethering to the OSC *flamenco* promoter. Data are shown for *cluster 20A* (C), *cluster 77B* (D), the *myc* 3′ UTR (E), and the *tj* 3′ UTR (F). (**G**) 1-kb tile analysis of fold changes in small RNA levels upon repressor tethering to the OSC *flamenco* promoter, shown for the entire OSC_r1.01 X chromosome. The extended OSC *flamenco* locus spanning the region from *DIP1* to *CG14621* is indicated as shaded area. (**H**) Log fold change in antisense TE-mapping piRNA levels upon *flamenco* silencing relative to control conditions. (**I**) Genome browser view of the OSC_r1.01 *flamenco* locus including the upstream *DIP1* gene. All identified splice junctions within the *flamenco* region are shown. Splicing efficiency was calculated as the percentage of spliced reads relative to the average read coverage within the preceding 5 nt. Analyzable regions are indicated, whereas regions excluded due to genome multi-mapping were not assessed for splice junctions. RNA-seq coverage of the reads used for splice analysis is shown as counts per million mapped reads.

SUPPLEMENTARY TABLES

Table S1 - Loss of heterozygosity (LOH) regions in OSCs.

BED coordinates of LOH regions as detected in Figure 1H.

CHR	START	STOP	LOH_ID
2L_RagTag	5000	1979000	LOH_1_1974000
2L_RagTag	5512000	8944000	LOH_2_3432000
2L_RagTag	9348000	11049000	LOH_3_1701000
2L_RagTag	15758000	16642000	LOH_4_884000
2L_RagTag	17742000	24705006	LOH_5_6963006
2R_RagTag	0	25983000	LOH_6_25983000
3L_RagTag	22000	8798000	LOH_7_8776000
3L_RagTag	22137000	22671000	LOH_8_534000
3L_RagTag	22671000	23215000	LOH_9_544000
3L_RagTag	28775000	29334000	LOH_10_559000
3R_RagTag	27313000	34926000	LOH_11_7613000
X_RagTag	0	27681000	LOH_12_27681000

Table S2 – Data used in this study (Additional File 2)

Comprehensive list of all next-generation sequencing (NGS) datasets analyzed in this manuscript, including both newly generated data and publicly available datasets. The table provides specific accession numbers, library types, and source information for each dataset.

Table S3 – Key Resources

REAGENT or RESOURCE	SOURCE	IDENTIFIER
Amaxa 4D Nucleofector Buffer SF	Lonza	V4XC-2024
Amaxa Nucleofector Kit V	Lonza	VCA-1003
AMPure XP beads	Beckman Coulter	AM63881
biotin-11-CTP	Perkin Elmer	NEL542001EA
biotin-11-UTP	Perkin Elmer	NEL543001EA
biotin-14-dATP (1 mM)	Jena Bioscience	NU-835-BIO14-S
Blasticidin	Invitrogen	A1113903
BSA (10 mg/mL)	New England Biolabs	B9001
Chloroform	Sigma Aldrich	25666
Covaris microTUBEs with AFA fibers	Covaris	520052
DpnII restriction enzyme	New England Biolabs	R0543L
Dynabeads MyOne Streptavidin C1	Thermo Fisher Scientific	65002
Dynabeads oligo(dT)	Thermo Fisher Scientific	61005
Ethanol (100%)	Sigma Aldrich	32205
EvaGreen dye	Biotium	3100
Fetal Bovine Serum (FBS)	VWR	422351S
Fly extract	Homemade according to [1]	N/A
Formaldehyde (16%)	Thermo Fisher Scientific	28908
Formamide (95%)	Merck Chemicals	344206
Glutathione	Sigma Aldrich	G6013
GlycoBlue coprecipitant	Thermo Fisher Scientific	AM9515
Glycogen	N/A	N/A
Insulin	Sigma Aldrich	I9278
Isopropanol	VWR	20842.312

KAPA HiFi polymerase	Roche	KK2602
Klenow fragment	New England Biolabs	M0210L
Low-melt agarose	Sigma Aldrich	A9414
LSK108 Ligation Sequencing Kit	Oxford Nanopore Technologies	SQK-LSK108
M3 basal medium	USbio	S1013
M3 basal medium	Sigma Aldrich	S3652
MaXtract High Density tubes	Qiagen	129073
NEB buffer 2	New England Biolabs	B7002S
NEB buffer 3.1	New England Biolabs	B7203S
NEBNext DNA Library Prep Kit	New England Biolabs	E730L
NEBNext Illumina Adaptor	New England Biolabs	Part of E6444S
NEBNext Ultra II Directional RNA Library Prep Kit	New England Biolabs	E7420S
NEBNext® Ultra™ II End Repair/dA-Tailing Module	New England Biolabs	E7546S
NP40 substitute	VWR	AMREM158-500
Nuclease-free water	Ambion	AM9937
P30 columns	Bio-Rad	7326250
Phenol (pH 8.0)	Sigma Aldrich	P3803
Phosphate-Buffered Saline (PBS)	Homemade (NaCl 8g/L; KCl 0.2 g/L; Na2HPO4 1.15 g/L; KH2PO4 0.2 g/L)	N/A
Protease inhibitors	Roche	04693132001
Proteinase K (20 mg/mL)	Sigma Aldrich	3115879001
Puregene Proteinase K	Qiagen	19157
Puromycin	Invitrogen	A1113803
Qubit Assay Kit DNA BR	Thermo Fisher Scientific	Q32853
Qubit Assay Kit RNA BR	Thermo Fisher Scientific	Q10211
R9.4 Flow Cell (FLO-MIN106)	Oxford Nanopore Technologies	FLO-MIN106
RAD002 Rapid Sequencing Kit	Oxford Nanopore Technologies	SQK-RAD002
RNA002 Direct RNA Sequencing Kit	Oxford Nanopore Technologies	SQK-RNA002
RNase A solution	Qiagen	19101
RNaseOUT	Thermo Fisher Scientific	10777019
SuperScript II Reverse Transcriptase	Thermo Fisher Scientific	18064014
T4 DNA ligase	Thermo Fisher Scientific	EL0011
T4 Polynucleotide Kinase (PNK)	New England Biolabs	M0201L
T4 RNA ligase 1	New England Biolabs	M0204L

T4 RNA ligase 2, truncated KQ	New England Biolabs	M0373L
TAP (Tobacco Acid Pyrophosphatase)	Epicentre	T19500
Thermostable Hybridase RNase H	Epicentre/Lucigen	108211
TraPR resin columns	Homemade according to [2]	N/A
Triton X-100 (10%)	Sigma Aldrich	X100-500ML
Trizol	Thermo Fisher Scientific	15596018
Trizol-LS	Thermo Fisher Scientific	10296028
Turbo DNase	Thermo Fisher Scientific	AM1907
Tween-20	Sigma Aldrich	P2287
Urea Polyacrylamide gel	National Diagnostics	EC833
USER enzyme	New England Biolabs	M5505L
Zymo DNA Clean & Concentrator-5	Zymo Research	D4014
Zymo Gel DNA Recovery Kit	Zymo Research	D4008
Zymo RNA Clean & Concentrator-5	Zymo Research	R1016
Zymo ZR small RNA PAGE Recovery Kit	Zymo Research	R1070
Oligonucleotides		
CRISPR-Cas9 sgRNA targeting flamenco upstream region (target sequence: TATAAAAGTTACAAAATACG)	This study	N/A
3' adaptor with 6 random bases and sample barcode sRBC-1 (/5rApp/ NNNNNN CAGTG AGATCGGAAGAGCACACGTCT /3SpC3/)	This study	N/A

3' adaptor with 6 random bases and sample barcode sRBC-2 (/5rApp/ NNNNNN AGCAA AGATCGGAAGAGCACACGTCT /3SpC3/)	This study	N/A
3' adaptor with 6 random bases and sample barcode sRBC-3 (/5rApp/ NNNNNN GGTAT AGATCGGAAGAGCACACGTCT /3SpC3/)	This study	N/A
3' adaptor with 6 random bases and sample barcode sRBC-4 (/5rApp/ NNNNNN TACCA AGATCGGAAGAGCACACGTCT /3SpC3/)	This study	N/A
3' adaptor with 6 random bases and sample barcode sRBC-5 (/5rApp/ NNNNNN GTCAG AGATCGGAAGAGCACACGTCT /3SpC3/)	This study	N/A
3' adaptor with 6 random bases and sample barcode sRBC-6 (/5rApp/ NNNNNN TGACT AGATCGGAAGAGCACACGTCT /3SpC3/)	This study	N/A
3' adaptor with 6 random bases and sample barcode sRBC-7 (/5rApp/ NNNNNN CGTTC AGATCGGAAGAGCACACGTCT /3SpC3/)	This study	N/A

3' adaptor with 6 random bases and sample barcode sRBC-8 (/5rApp/ NNNNNN ATGGA AGATCGGAAGAGCACACGTCT /3SpC3/)	This study	N/A
3' adaptor with 6 random bases and sample barcode sRBC-9 (/5rApp/ NNNNNN GAACG AGATCGGAAGAGCACACGTCT /3SpC3/)	This study	N/A
3' adaptor with 6 random bases and sample barcode sRBC-10 (/5rApp/ NNNNNN ACGAG AGATCGGAAGAGCACACGTCT /3SpC3/)	This study	N/A
3' adaptor with 6 random bases and sample barcode sRBC-11 (/5rApp/ NNNNNN CTGAT AGATCGGAAGAGCACACGTCT /3SpC3/)	This study	N/A
3' adaptor with 6 random bases and sample barcode sRBC-12 (/5rApp/ NNNNNN TCCAT AGATCGGAAGAGCACACGTCT /3SpC3/)	This study	N/A
Fluorescent oligo short for sRNAseq (/5IRD800CWN/AGTCGCATAGATTCATAGCCTTGTGGAGTCTGTAGAGGGAATTGT/3ddC/)	This study	N/A

Fluorescent oligo long for sRNAseq (/5IRD800CWN/AGTCGCATAGATTCATAGCCTTGTGGAGTCTGTAGAGGGAATTGTTATTGCAGAACTGGTTACTGA/3ddC/)	This study	N/A
5' adaptor RNA oligo with 4 random nucleotides at 3' terminus (ACACUCUUUCCCUACACGACGCUCUUCCGAUCUNNNN; for sRNAseq and PROseq)	[3]	N/A
3' adaptor with 4 random nucleotides at 5' end (for PROseq; /5rApp/NNNNAGATCGGAAGAGCACACGTCT/3ddC/)	[3]	N/A
DNA oligos complementary to rRNA (50-mer)	[4]	N/A

Recombinant DNA	***Plasmid maps of newly created plasmids available at:***	***https://github.com/BrenneckeLab/Handler_2026-OSC-genome/tree/main/PlasmidMaps***
Cas9 expression vector	Addgene	Cat# 49330; pAc-sgRNA-Cas9
phiC31 integrase expression plasmid	Addgene	Cat# 26290; pBS130
Donor repair template with attP-flanked Puro-mCherry cassette	This study	LandingSite_Repair_Template.gbk
attB-mCherry-Blasticidin-14xUAS-attB plasmid	This study	LandingSite_CasseteExchange_14xUAS.gbk
Gal4 expression plasmid	[5]	N/A
Gal4-Panoramix IDR silencing domain fusion plasmid	[5]	N/A

Table S4 – Software Reference table

Name	Version	Reference	Description of use	Category
**minimap2**	2.18/2.24	[6]	Oxford Nanopore read alignment (DNA and RNA); splice-aware mapping for direct RNA-seq	Bioinformatics - Alignment
**BWA-MEM**	0.7.17-r1188	[7]	Illumina short read alignment for Hi-C scaffolding and variant calling	Bioinformatics - Alignment
**Bowtie**	1.3.1	[8]	K-mer uniqueness mapping for genome mappability profiling	Bioinformatics - Alignment
**HISAT2**	2.2.1	[9]	Illumina RNA-seq read alignment for de novo transcript assembly	Bioinformatics - Alignment
**STAR**	2.7.10a	[10]	Short element alignment (<50 bp UTRs) and RNA-seq read mapping	Bioinformatics - Alignment
**BLAT**	39x1	[11]	Full-length transcript and CDS alignment to genome assembly	Bioinformatics - Alignment
**Shasta**	Commit-ID: 3367f960	[12]	De novo genome assembly from Oxford Nanopore long reads	Bioinformatics - Assembly
**wtdbg2/WTPOA**	2.5	[13]	De novo assembly for gap-filling contigs; TE sequence assembly	Bioinformatics - Assembly
**racon**	1.4.22	[14]	Consensus polishing of gap-filling contigs and assembled TE sequences	Bioinformatics - Assembly
**Purge Haplotigs**	Modified from V1.1.1 (modified version vDH_1.0 available: https://gitlab.com/dominik-handler/purgehaplotigs)	[15]	Identification and removal of redundant haplotigs from assembly	Bioinformatics - Assembly QC
**BUSCO**		[16]	Assessment of genome assembly completeness using single-copy orthologs	Bioinformatics - Assembly QC
**SALSA2**	cf0fa8e	[17]	Hi-C-based scaffolding of genome assembly; DpnII restriction enzyme motif	Bioinformatics - Scaffolding
**RagTag**	2.1.0	[18]	Super-scaffolding against dm6 reference genome	Bioinformatics - Scaffolding
**Clair3**	0.1-r12	[19]	SNV and indel calling from ONT reads; first-round polishing	Bioinformatics - Variant Calling
**PEPPER-Margin-DeepVariant**	0.7.0	[20]	Second-round polishing with ONT reads; variant calling pipeline	Bioinformatics - Variant Calling
**DeepVariant**	1.1.0	[21]	Illumina short-read variant calling; final polishing step; SNP/indel detection	Bioinformatics - Variant Calling
**Sniffles**	1.012a	[22]	Detection of missing TEs in the primary assembly before re-insertion	Bioinformatics - Variant Calling
**Sniffles**	2.	[23]	Structural variant detection from ONT long reads; TE insertion identification	Bioinformatics - Variant Calling
**cuteSV**	1.0.13	[24]	Structural variant detection from ONT reads	Bioinformatics - Variant Calling
**RepeatMasker**	4.1.0/4.1.2	[25]	Transposable element and repeat annotation across genome assembly	Bioinformatics - Annotation
**Dfam**	3.1	[26]	Repeat family database for RepeatMasker annotations	Bioinformatics - Annotation
**Liftoff**	1.6.3	[27]	Transfer of dm6 gene annotations to OSC genome assembly	Bioinformatics - Annotation

**StringTie**	2.2.1	[28]	De novo transcript assembly from RNA-seq data	Bioinformatics - RNA-seq
**Guppy**	5.0.7+2332e8d65	Oxford Nanopore Technologies	GPU-accelerated base-calling for ONT DNA and direct RNA sequencing with super-accuracy model	Bioinformatics - Base calling
**Porechop**	0.2.4	[29]	Adapter trimming from Oxford Nanopore reads	Bioinformatics - Preprocessing
**Yacrd**	0.6.2	[30]	Read scrubbing to remove chimeric sequences from ONT reads	Bioinformatics - Preprocessing
**Filtlong**	0.2.0	[31]	Quality and length filtering of ONT reads for assembly	Bioinformatics - Preprocessing
**tailfindr**	0.1.0	[32]	Poly(A) tail identification and length estimation from direct RNA-seq	Bioinformatics - Preprocessing
**samtools**	1.14	[33]	BAM file manipulation, sorting, indexing, filtering, and duplicate marking	Bioinformatics - Utilities
**bcftools**	1.14	[33]	Variant file processing; consensus sequence generation from VCF	Bioinformatics - Utilities
**bedtools**	v2.28.0	[34]	Genomic interval operations; coverage calculation; BED/BAM conversions	Bioinformatics - Utilities
**seqkit**	2.3.0	[35]	Sequence manipulation; sliding-window k-mer generation; ID sanitization	Bioinformatics - Utilities
**Picard**		[36]	Read group assignment; PCR duplicate marking for Hi-C and variant calling	Bioinformatics - Utilities
**rtg-tools**	3.13	[37]	VCF statistics and variant file analysis (vcfstats)	Bioinformatics - Utilities
**UCSC Kent Utilities**		[38]	faToTwoBit conversion; bigBed/bigWig generation; hub construction	Bioinformatics - Genome Browser
**paf2dotplot**	f8dc552	[39]	Dot plot visualization of genome alignments from minimap2 PAF output	Bioinformatics - Visualization
**CHOPCHOP**	V2	[40, 41]	CRISPR sgRNA design for landing-site cell line generation	Bioinformatics - CRISPR
**Apptainer**	1.1.9-1	[42, 43]	Container management for reproducible pipeline execution	Infrastructure - Containerization
**NanoPlot**		[44]	Quality assessment and visualization of ONT long-read sequencing data	Bioinformatics - QC
**QUAST**	5.2.0, 2723558f	[45]	Genome assembly quality assessment and comparison	Bioinformatics - Assembly QC
**GAEP**	1.2.3	[46]	Comprehensive genome assembly evaluation pipeline	Bioinformatics - Assembly QC
**Flye**	2.9-b1774	[47]	De novo assembly of long reads; TE sequence assembly	Bioinformatics - Assembly
**pairtools**	1.1.3	[48]	Hi-C pair-end read processing and filtering	Bioinformatics - Hi-C
**cooler**	0.8.10	[49]	Hi-C contact matrix storage and manipulation	Bioinformatics - Hi-C
**cooltools**	0.3.2	[50]	Hi-C analysis toolkit for contact frequency analysis	Bioinformatics - Hi-C
**SyRI**	1.6.3	[51]	Identification of structural rearrangements and synteny blocks	Bioinformatics - Synteny
**plotsr**	1.1.1	[52]	Visualization of synteny and structural rearrangements	Bioinformatics - Visualization

**Dot**		[53]	Dot plot preparation for genome comparison visualization	Bioinformatics - Visualization
**MUMmer**		[54]	Whole genome alignment (nucmer, delta-filter, show-coords)	Bioinformatics - Alignment
**gffread**		[55]	GFF/GTF file processing and conversion	Bioinformatics - Annotation
**BEDOPS**	2.4.38	[56]	Genomic interval operations	Bioinformatics - Utilities
**regtools**	1.0.0	[57]	RNA-seq junction extraction and annotation	Bioinformatics - RNA-seq
**tabix**			Indexing and rapid retrieval of tab-delimited genomic data	Bioinformatics - Utilities
**GNU Parallel**	20250722	[58]	Parallelization of command-line tools for efficient processing	Infrastructure - Utilities
**R**	4.3.2		Statistical computing and graphics	Statistics - Software
**tidyverse**	2.0.0	[59]	Data manipulation and visualization (includes ggplot2, dplyr)	Statistics - R packages
**cowplot**	1.1.3	[60]	Publication-ready figure composition	Statistics - R packages
**patchwork**	1.3.0	[61]	Combining multiple ggplot2 plots	Statistics - R packages
**ggalluvial**	0.12.5	[62]	Alluvial diagram visualization	Statistics - R packages
**dplyr**	1.1.4	[63]	Data manipulation and transformation (part of tidyverse)	Statistics - R packages
**ggbeeswarm**	0.7.2	[64]	Beeswarm-style categorical scatter plots to avoid overplotting	Statistics - R packages
**ggforce**	0.4.2	[65]	Extended ggplot2 functionality with additional geoms and stats	Statistics - R packages
**ggh4x**	0.3.1	[66]	Extended faceting and axis customization for ggplot2	Statistics - R packages
**ggrastr**	1.0.2	[67]	Rasterization of ggplot2 layers for large datasets	Statistics - R packages
**jsonlite**	1.9.0	[68]	JSON parsing and generation for data import/export	Statistics - R packages
**khroma**	1.16.0	[69]	Color-blind friendly color schemes for scientific visualization	Statistics - R packages
**paletteer**	1.6.0	[70]	Unified access to color palettes from multiple R packages	Statistics - R packages
**scales**	1.3.0	[71]	Scale functions for data visualization (axis breaks, labels, transformations)	Statistics - R packages
**readr**	2.1.5	[72]	Fast and friendly reading of rectangular data (part of tidyverse)	Statistics - R packages

SUPPLEMENTARY REFERENCES

1. Saito K, Inagaki S, Mituyama T, Kawamura Y, Ono Y, Sakota E, Kotani H, Asai K, Siomi H, Siomi MC: **A regulatory circuit for piwi by the large Maf gene traffic jam in Drosophila.** *Nature* 2009, **461:**1296-1299.
2. Grentzinger T, Oberlin S, Schott G, Handler D, Svozil J, Barragan-Borrero V, Humbert A, Duharcourt S, Brennecke J, Voinnet O: **A universal method for the rapid isolation of all known classes of functional silencing small RNAs.** *Nucleic Acids Res* 2020, **48:**e79.
3. Jayaprakash AD, Jabado O, Brown BD, Sachidanandam R: **Identification and remediation of biases in the activity of RNA ligases in small-RNA deep sequencing.** *Nucleic Acids Research* 2011, **39:**e141-e141.
4. Batki J, Schnabl J, Wang J, Handler D, Andreev VI, Stieger CE, Novatchkova M, Lampersberger L, Kauneckaite K, Xie W, et al: **The nascent RNA binding complex SFiNX licenses piRNA-guided heterochromatin formation.** *Nat Struct Mol Biol* 2019, **26:**720-731.
5. Andreev VI, Yu C, Wang J, Schnabl J, Tirian L, Gehre M, Handler D, Duchek P, Novatchkova M, Baumgartner L, et al: **Panoramix SUMOylation on chromatin connects the piRNA pathway to the cellular heterochromatin machinery.** *Nat Struct Mol Biol* 2022, **29:**130-142.
6. Li H: **Minimap2: pairwise alignment for nucleotide sequences.** *Bioinformatics* 2018, **34:**3094-3100.
7. Li H: **Aligning sequence reads, clone sequences and assemblycontigs with BWA-MEM.** arXiv.org; 2013.
8. Langmead B, Trapnell C, Pop M, Salzberg SL: **Ultrafast and memory-efficient alignment of short DNA sequences to the human genome.** *Genome Biol* 2009, **10:**R25.
9. Kim D, Paggi JM, Park C, Bennett C, Salzberg SL: **Graph-based genome alignment and genotyping with HISAT2 and HISAT-genotype.** *Nat Biotechnol* 2019, **37:**907-915.
10. Dobin A, Davis CA, Schlesinger F, Drenkow J, Zaleski C, Jha S, Batut P, Chaisson M, Gingeras TR: **STAR: ultrafast universal RNA-seq aligner.** *Bioinformatics* 2013, **29:**15-21.
11. Kent WJ: **BLAT--the BLAST-like alignment tool.** *Genome Res* 2002, **12:**656-664.
12. Shafin K, Pesout T, Lorig-Roach R, Haukness M, Olsen HE, Bosworth C, Armstrong J, Tigyi K, Maurer N, Koren S, et al: **Nanopore sequencing and the Shasta toolkit enable efficient de novo assembly of eleven human genomes.** *Nat Biotechnol* 2020, **38:**1044-1053.
13. Ruan J, Li H: **Fast and accurate long-read assembly with wtdbg2.** *Nat Methods* 2020, **17:**155-158.
14. Vaser R, Sović I, Nagarajan N, Šikić M: **Fast and accurate de novo genome assembly from long uncorrected reads.** *Genome Research* 2017, **27:**737-746.

15. Roach MJ, Schmidt SA, Borneman AR: **Purge Haplotigs: allelic contig reassignment for third-gen diploid genome assemblies.** *BMC Bioinformatics* 2018, **19:**460.
16. Manni M, Berkeley MR, Seppey M, Simao FA, Zdobnov EM: **BUSCO Update: Novel and Streamlined Workflows along with Broader and Deeper Phylogenetic Coverage for Scoring of Eukaryotic, Prokaryotic, and Viral Genomes.** *Mol Biol Evol* 2021, **38:**4647-4654.
17. Ghurye J, Rhie A, Walenz BP, Schmitt A, Selvaraj S, Pop M, Phillippy AM, Koren S: **Integrating Hi-C links with assembly graphs for chromosome-scale assembly.** *PLoS Comput Biol* 2019, **15:**e1007273.
18. Alonge M, Lebeigle L, Kirsche M, Jenike K, Ou S, Aganezov S, Wang X, Lippman ZB, Schatz MC, Soyk S: **Automated assembly scaffolding using RagTag elevates a new tomato system for high-throughput genome editing.** *Genome Biol* 2022, **23:**258.
19. Zheng Z, Li S, Su J, Leung AW, Lam TW, Luo R: **Symphonizing pileup and full-alignment for deep learning-based long-read variant calling.** *Nat Comput Sci* 2022, **2:**797-803.
20. Shafin K, Pesout T, Chang PC, Nattestad M, Kolesnikov A, Goel S, Baid G, Kolmogorov M, Eizenga JM, Miga KH, et al: **Haplotype-aware variant calling with PEPPER-Margin-DeepVariant enables high accuracy in nanopore long-reads.** *Nat Methods* 2021, **18:**1322-1332.
21. Poplin R, Chang PC, Alexander D, Schwartz S, Colthurst T, Ku A, Newburger D, Dijamco J, Nguyen N, Afshar PT, et al: **A universal SNP and small-indel variant caller using deep neural networks.** *Nat Biotechnol* 2018, **36:**983-987.
22. Sedlazeck FJ, Rescheneder P, Smolka M, Fang H, Nattestad M, von Haeseler A, Schatz MC: **Accurate detection of complex structural variations using single-molecule sequencing.** *Nat Methods* 2018, **15:**461-468.
23. Smolka M, Paulin LF, Grochowski CM, Horner DW, Mahmoud M, Behera S, Kalef-Ezra E, Gandhi M, Hong K, Pehlivan D, et al: **Detection of mosaic and population-level structural variants with Sniffles2.** *Nat Biotechnol* 2024, **42:**1571-1580.
24. Jiang T, Liu Y, Jiang Y, Li J, Gao Y, Cui Z, Liu Y, Liu B, Wang Y: **Long-read-based human genomic structural variation detection with cuteSV.** *Genome Biol* 2020, **21:**189.
25. Smit AH, R;Green, P: **RepeatMasker Open-4.0.** 2013-2015.
26. Storer J, Hubley R, Rosen J, Wheeler TJ, Smit AF: **The Dfam community resource of transposable element families, sequence models, and genome annotations.** *Mob DNA* 2021, **12:**2.
27. Shumate A, Salzberg SL: **Liftoff: accurate mapping of gene annotations.** *Bioinformatics* 2021, **37:**1639-1643.
28. Shumate A, Wong B, Pertea G, Pertea M: **Improved transcriptome assembly using a hybrid of long and short reads with StringTie.** *PLoS Comput Biol* 2022, **18:**e1009730.
29. Wick RR, Judd LM, Gorrie CL, Holt KE: **Completing bacterial genome assemblies with multiplex MinION sequencing.** *Microb Genom* 2017, **3:**e000132.

30. Marijon P, Chikhi R, Varre JS: **yacrd and fpa: upstream tools for long-read genome assembly.** *Bioinformatics* 2020, **36:**3894-3896.
31. Wick RR: **FiltLong**. https://github.com/rrwick/Filtlong, (2018)
32. Krause M, Niazi AM, Labun K, Torres Cleuren YN, Muller FS, Valen E: **tailfindr: alignment-free poly(A) length measurement for Oxford Nanopore RNA and DNA sequencing.** *RNA* 2019, **25:**1229-1241.
33. Danecek P, Bonfield JK, Liddle J, Marshall J, Ohan V, Pollard MO, Whitwham A, Keane T, McCarthy SA, Davies RM, Li H: **Twelve years of SAMtools and BCFtools.** *Gigascience* 2021, **10**.
34. Quinlan AR, Hall IM: **BEDTools: a flexible suite of utilities for comparing genomic features.** *Bioinformatics* 2010, **26:**841-842.
35. Shen W, Sipos B, Zhao L: **SeqKit2: A Swiss army knife for sequence and alignment processing.** *Imeta* 2024, **3:**e191.
36. Institute B: **Picard Toolkit**. https://broadinstitute.github.io/picard/, (2019)
37. Genomics RT: **RTG Tools**. https://github.com/RealTimeGenomics/rtg-tools, (2018)
38. Perez G, Barber GP, Benet-Pages A, Casper J, Clawson H, Diekhans M, Fischer C, Gonzalez JN, Hinrichs AS, Lee CM, et al: **The UCSC Genome Browser database: 2025 update.** *Nucleic Acids Res* 2025, **53:**D1243-D1249.
39. Wilkie GS, Zimyanin V, Kirby R, Korey C, Francis-Lang H, Van Vactor D, Davis I: **Small bristles, the Drosophila ortholog of NXF-1, is essential for mRNA export throughout development.** *RNA (New York)* 2001, **7:**1781-1792.
40. Labun K, Montague TG, Krause M, Torres Cleuren YN, Tjeldnes H, Valen E: **CHOPCHOP v3: expanding the CRISPR web toolbox beyond genome editing.** *Nucleic Acids Res* 2019, **47:**W171-W174.
41. Labun K, Montague TG, Gagnon JA, Thyme SB, Valen E: **CHOPCHOP v2: a web tool for the next generation of CRISPR genome engineering.** *Nucleic Acids Res* 2016, **44:**W272-276.
42. Developers S: **Singularity.** *Zenodo* 2021.
43. Kurtzer GM, Sochat V, Bauer MW: **Singularity: Scientific containers for mobility of compute.** *PLoS One* 2017, **12:**e0177459.
44. De Coster W, Rademakers R: **NanoPack2: population-scale evaluation of long-read sequencing data.** *Bioinformatics* 2023, **39**.
45. Mikheenko A, Prjibelski A, Saveliev V, Antipov D, Gurevich A: **Versatile genome assembly evaluation with QUAST-LG.** *Bioinformatics* 2018, **34:**i142-i150.
46. Zhang Y, Lu HW, Ruan J: **GAEP: a comprehensive genome assembly evaluating pipeline.** *J Genet Genomics* 2023, **50:**747-754.
47. Kolmogorov M, Yuan J, Lin Y, Pevzner PA: **Assembly of long, error-prone reads using repeat graphs.** *Nat Biotechnol* 2019, **37:**540-546.
48. Abdennur N, Fudenberg G, Flyamer IM, Galitsyna AA, Goloborodko A, Imakaev M, Venev SV: **Pairtools: from sequencing data to chromosome contacts.** Cold Spring Harbor Laboratory; 2023.
49. Abdennur N, Mirny LA: **Cooler: scalable storage for Hi-C data and other genomically labeled arrays.** *Bioinformatics* 2020, **36:**311-316.

50. Open2C, Abdennur N, Abraham S, Fudenberg G, Flyamer IM, Galitsyna AA, Goloborodko A, Imakaev M, Oksuz BA, Venev SV, Xiao Y: **Cooltools: Enabling high-resolution Hi-C analysis in Python.** *PLoS Comput Biol* 2024, **20:**e1012067.
51. Goel M, Sun H, Jiao WB, Schneeberger K: **SyRI: finding genomic rearrangements and local sequence differences from whole-genome assemblies.** *Genome Biol* 2019, **20:**277.
52. Goel M, Schneeberger K: **plotsr: visualizing structural similarities and rearrangements between multiple genomes.** *Bioinformatics* 2022, **38:**2922-2926.
53. Nexus D: **Dot**. https://github.com/dnanexus/dot, (2019)
54. Marcais G, Delcher AL, Phillippy AM, Coston R, Salzberg SL, Zimin A: **MUMmer4: A fast and versatile genome alignment system.** *PLoS Comput Biol* 2018, **14:**e1005944.
55. gpertea: **gffread**. https://github.com/gpertea/gffread,
56. Neph S, Kuehn MS, Reynolds AP, Haugen E, Thurman RE, Johnson AK, Rynes E, Maurano MT, Vierstra J, Thomas S, et al: **BEDOPS: high-performance genomic feature operations.** *Bioinformatics* 2012, **28:**1919-1920.
57. Cotto KC, Feng YY, Ramu A, Richters M, Freshour SL, Skidmore ZL, Xia H, McMichael JF, Kunisaki J, Campbell KM, et al: **Integrated analysis of genomic and transcriptomic data for the discovery of splice-associated variants in cancer.** *Nat Commun* 2023, **14:**1589.
58. Tange O: **GNU Parallel 20250722 ('Fordow')**. https://doi.org/10.5281/zenodo.16289600, 10.5281/zenodo.16289600; (2025)
59. Wickham H, Averick M, Bryan J, Chang W, McGowan L, François R, Grolemund G, Hayes A, Henry L, Hester J, et al: **Welcome to the Tidyverse.** *Journal of Open Source Software* 2019, **4:**1686.
60. Wilke CO: **cowplot: Streamlined Plot Theme and Plot Annotations for 'ggplot2'**. https://CRAN.R-project.org/package=cowplot, (2024)
61. Pedersen TL: **patchwork: The Composer of Plots**. https://CRAN.R-project.org/package=patchwork, (2024)
62. Jason Cory Brunson QDR: **ggalluvial: Alluvial Plots in 'ggplot2'**. http://corybrunson.github.io/ggalluvial/, (2023)
63. Wickham H, François R, Henry L, Müller K, Vaughan D: **dplyr: A Grammar of Data Manipulation**. https://CRAN.R-project.org/package=dplyr, (2023)
64. Clarke E, Sherrill-Mix S, Dawson C: **ggbeeswarm: Categorical Scatter (Violin Point) Plots**. https://CRAN.R-project.org/package=ggbeeswarm, (2023)
65. Pedersen TL: **ggforce: Accelerating 'ggplot2'**. https://CRAN.R-project.org/package=ggforce, (2024)
66. Brand Tvd: **ggh4x: Hacks for 'ggplot2'**. https://CRAN.R-project.org/package=ggh4x, (2025)
67. Petukhov V, van den Brand T, Biederstedt E: **ggrastr: Rasterize Layers for 'ggplot2'**. https://CRAN.R-project.org/package=ggrastr, (2023)
68. J O: **The jsonlite Package: A Practical and Consistent Mapping Between JSON Data and R Objects.**, https://arxiv.org/abs/1403.2805, (2014)

69. Frerebeau N: **khroma: Colour Schemes for Scientific Data Visualization**. https://packages.tesselle.org/khroma/, (2025)
70. Hvitfeldt E: **paletteer: Comprehensive Collection of Color Palettes.**, https://github.com/EmilHvitfeldt/paletteer, (2021)
71. Wickham H, Pedersen TL, Seidel D: **scales: Scale Functions for Visualization**. https://CRAN.R-project.org/package=scales, (2023)
72. Hadley Wickham JH, Jennifer Bryan: **readr: Read Rectangular Text Data**. https://CRAN.R-project.org/package=readr, (2023)
